# Exploration of Novel Biomarkers Through a Precision Medicine Approach Using Multi‐Omics and Brain Organoids in Patients With Atypical Depression and Psychotic Symptoms

**DOI:** 10.1002/advs.202508383

**Published:** 2025-10-31

**Authors:** Insook Ahn, Soyeon Chang, Jiyoung Lee, Seok‐Ho Choi, Jinju Han, Yangsik Kim

**Affiliations:** ^1^ Graduate School of Medical Science and Engineering Korea Advanced Institute of Science and Technology Daejeon 34051 Republic of Korea; ^2^ Department of Psychiatry Inha University Hospital, College of Medicine, Inha University Incheon 22332 Republic of Korea

**Keywords:** brain organoid, major depression disorder, psychotic disorder, proteomics, single‐cell transcriptomics

## Abstract

Major depressive disorder (MDD) with atypical features accompanied by psychotic symptoms represents a severe and under‐researched subtype of depression and severe mental illness, characterized by significant personal and social impact. This study aims to explore novel biomarkers through a precision medicine approach by combining clinical data, white blood cell (WBC) single‐cell RNA sequencing (scRNA‐seq), plasma proteomics, and brain organoid models to uncover immunological and neurological alterations in patients with this condition. Patients exhibited elevated stress, anxiety, depression, and increased WBC counts, although the difference in WBC count is not significant after adjusting for age. Plasma proteomic profiling identified an upregulation of proteins implicated in synaptic formation, including Doublecortin‐Like Kinase 3 (DCLK3) and Calcyon (CALY), as well as immune‐related proteins such as Complement Component 5 (C5). WBC scRNA‐seq revealed significant neutrophil and monocyte transcriptomic alterations, suggesting increased inflammation and immune dysregulation. Patient‐derived brain organoids display reduced growth and distinct gene expression patterns compared to controls, particularly under dexamethasone‐induced stress conditions. Combining WBC scRNA‐seq, plasma proteomics, and brain organoid models offers a novel framework for understanding the pathophysiology of psychiatric disorders, which is one of the most complex disorders.

## Graphical abstract

This study examines major depressive disorder with atypical features and psychotic symptoms through a multi‐omics precision medicine approach. By integrating clinical assessments, WBC scRNA‐seq, plasma proteomics, and brain organoid models, it uncovers immune dysregulation, synaptic protein alterations, and stress‐sensitive neuronal changes. The results offer insights into disease mechanisms and identify potential biomarkers for targeted therapeutic strategies in severe mental illness.

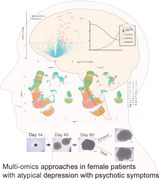

## 1 Introduction

The Global Burden of Disease 2019 showed that mental disorders affected 907.1 million people and accounted for 125.3 million disability‐adjusted life years (DALYs), ranking it as the seventh highest contributor to the global disease burden.^[^ ^1^ ^]^ Mood disorders, following anxiety disorders, affected 279.6 million people (28.8%), with a higher prevalence among women, and accounted for the highest proportion of DALYs (37.3%). Major depressive disorder (MDD), which is one of the most common mood disorders, is classified as treatment‐resistant depression in about one‐third of cases and causes significant personal distress and social burden.^[^ ^2^ ^]^ Despite the high prevalence and social impact of mood disorders, there is a lack of objective biomarkers for their diagnosis and treatment, highlighting the need for further research.^[^ ^3^ ^]^

Immunological abnormalities have been consistently reported in psychiatric disorders, particularly MDD.^[^ ^3^, ^4^, ^5^, ^6^, ^7^, ^8^, ^9^, ^10^, ^11^ ^]^ MDD with immune dysfunction is often less responsive to conventional antidepressant medication use, and patients with distinct immunological abnormalities have shown improvement with the use of infliximab, an anti‐TNF immunosuppressant.^[^ ^12^ ^]^ It is also well established that depressive symptoms can be induced by the pro‐inflammatory cytokine such as IFN‐γ.^[^ ^13^ ^]^ Among the MDD subtypes, patients with atypical and melancholic features reportedly exhibit immunological abnormalities.^[^ ^6^, ^7^, ^8^, ^9^, ^14^, ^15^, ^16^ ^]^ These specific subtypes may exhibit distinct immune activation profiles (such as Th1 and Th2 responses), potentially necessitating tailored treatment strategies, although clinical studies have yet to demonstrate significant differences in drug efficacy based on these subtypes.^[^ ^17^, ^18^, ^19^, ^20^ ^]^ Among these, atypical depression is recognized as a biologically and clinically distinct form, accounting for up to 40% of all MDD cases, and is more common in women, often begins early in life, and frequently co‐occurs with bipolar II disorder.^[^ ^21^, ^22^, ^23^, ^24^ ^]^ These characteristics suggest underlying sex‐specific and neurobiological mechanisms. Accordingly, the present study focused on female patients with atypical depression to explore female‐specific pathophysiological features through multi‐omics profiling and organoid modeling.

Psychotic disorders, including schizophrenia, have also been associated with immunological abnormalities, which share biological features with mood disorders.^[^ ^25^, ^26^, ^27^, ^28^ ^]^ Common, non‐pathognomonic cytokine alterations have been observed in mood disorders and schizophrenia, ^[^ ^29^, ^30^ ^]^ and immune dysfunction has also been reported in studies of MDD accompanied by psychotic symptoms.^[^ ^31^, ^32^ ^]^ Mood and psychotic symptoms in adulthood are commonly associated with childhood stress, adversity, and inflammatory states.^[^ ^33^, ^34^, ^35^, ^36^ ^]^ Additionally, psychotic experiences, including idea of reference (IOR), are observed in ≈8% of the general population^[^ ^37^, ^38^ ^]^; psychotic symptoms occur in 9.8%– 10.92% of patients with MDD and are associated with more severe depression, higher anxiety levels, and increased suicide risk.^[^ ^39^, ^40^, ^41^, ^42^ ^]^ Thus, we focused on patients with severe mental illness (SMI), particularly those diagnosed with MDD with atypical features accompanied by psychotic symptoms, because this condition is associated with a more severe disease trajectory.

One of the greatest challenges in psychiatric research is the inability to study a living and functional brain that is intact and connected. Psychiatric disorders are triggered by the complex interplay of individual vulnerabilities and environmental stress, including lifetime adversity. These vulnerabilities encompass not only genetic predispositions but also acquired factors, such as stress‐induced changes, particularly recently reported brain somatic mosaicism.^[^ ^43^, ^44^ ^]^ Therefore, an approach that reflects both a functioning brain and an individual's biological characteristics is required, and these limitations, arising from a lack of representative models, could potentially be addressed using brain organoids.^[^ ^45^, ^46^, ^47^ ^]^ By isolating blood monocytes and using induced pluripotent stem cells (iPSCs) to differentiate and form brain organoids, an in vitro functional brain model can reflect patient genetic and biological characteristics. Additionally, to mimic stress‐related neuroendocrine responses, we exposed patient‐derived brain organoids to dexamethasone (DMX), ^[^ ^48^ ^]^ a synthetic glucocorticoid that mirrors the hormonal output of the hypothalamic‐pituitary‐adrenal (HPA) axis. This model provides a physiologically relevant framework to explore how stress hormones influence neural development and function in a patient‐specific context.

In this study, in addition to inflammatory markers such as C‐reaction protein (CRP) and interleukin (IL)‐6, which are routinely measured in clinical studies, we aimed to explore a broader range of biomarkers using plasma proteomics, WBC single‐cell transcriptomics, and patient‐derived brain organoid model. Furthermore, we focused on identifying biomarkers in female patients with MDD with atypical features accompanied by psychotic symptoms, an area that has been relatively under‐researched.

## 2 Results

### 2.1 Higher Stress Levels, Psychiatric Symptoms, and Elevated WBC Count in the Patient Group

This study compared 7 female patients with atypical depression and psychotic symptoms to 10 healthy female controls (**Table** **1**). The patient group was younger (29.1 years versus 37.4 years, *p* = 0.015), and only 1 of 7 patients was married, compared with 7 of 10 women in the control group (*p* = 0.074). In terms of employment, 2 of 7 patients were employed, while 9 of 10 participants in the control group were employed (*p* = 0.051). Physical examination and social history revealed that the pulse rate was significantly higher in the patient group (*p* = 0.040).

**Table 1:** Clinical Characteristics and Laboratory Results of Participants Adjusted for Age.

	Mean [SD] or n [%]		t‐statistic, U, or OR [CI]	*Cohen's d or Cliff's delta* [CI]	*p*	*p adj*
	Patients (*n* = 7)	Controls (*n* = 10)				
Age, years	29.1 (7.67)	37.4 (4.74)	−2.75	−1.36 [−14.65, −1.86]	*0.015	–
Married	1 (14.3%)	7 (70.0%)	14 [1.14 – 172.64]	−0.05 [−0.97, −0.15]	0.074	–
Employment	2 (28.6%)	9 (90.0%)	∞ [0.60 – 371.91]	−0.43 [−0.82, ‐0.03]	0.051	–
Smoking	3 (42.9%)	0	0 [0.002 – 1.65]	0.43 [0.03, 0.82]	0.051	–
Drinking	1 (14.3%)	4 (40%)	4 [0.34 – 47.11]	−0.26 [−0.68, 0.17]	0.338	–
SBP, mmHg	123.4 (14.00)	113.8 (12.96)	1.46	0.72 [−3.49, 22.75]	0.165	0.786
DBP, mmHg	79.1 (8.65)	71.8 (8.61)	1.73	0.85 [−1.0, 15.68]	0.105	0.453
PR, bpm	93.4 (20.74)	76.2 (10.80)	2.25	1.11 [0.47, 33.99]	*0.040	0.342
BW, kg^†^	82.6 (29.08)	66.7 (13.93)	47	0.34 [−7.26, 39.15]	0.270	0.808
Height, cm	163.1 (3.77)	162.5 (5.35)	0.24	0.12 [−3.76, 4.9]	0.812	0.951
Alcohol per week^†^	0.1 (0.38)	0.4 (0.70)	29	−0.17 [−0.77, 0.26]	0.469	0.730
Alcohol, bottle^†^	0.1 (0.38)	0.4 (0.52)	26	−0.26 [−0.68, 0.17]	0.294	0.701
Problematic drinking^†^	0.3 (0.76)	0.3 (0.48)	31	−0.11 [−0.65, 0.62]	0.645	0.726
Self‐rated Scales						
Stress visual scale	62.0 (19.96)	33.3 (22.47)	2.71	1.34 [8.39, 49.01]	*0.016	0.162
Suicidal idea^†^	1.0 (1.00)	0 (0)	60	0.71 [0.26, 1.74]	**0.003	0.091
SPQ‐IOR, total^†^	4.6 (0.98)	1.6 (0.97)	68	0.94 [2.03, 3.91]	**0.001	**0.003
LEC‐5 numbers	4.3 (3.04)	1.2 (1.03)	3.01	1.48 [0.74, 5.43]	**0.009	0.060
PSS	26.7 (6.32)	16.0 (5.27)	3.81	1.88 [5.01, 16.42]	**0.002	*0.047
HAM‐D^†^	22.7 (10.70)	1.1 (1.29)	70	1.00 [13.64, 29.58]	**0.001	**0.004
HAM‐A^†^	25.4 (10.97)	1.4 (1.51)	70	1.00 [15.85, 32.21]	**0.001	**0.001
Blood Tests								
WBC, × 10^9^/mm^3^	7.5 (2.40)	5.3 (1.71)	2.24	1.10 [0.15, 4.29]	*0.041	0.533
RBC, × 10^9^/mm^3^	12.4 (0.60)	12.7 (0.23)	1.79	0.88 [−0.1, 0.84]	0.094	0.361
Hb, g/dL	12.4 (2.33)	12.7 (0.97)	−0.32	−0.16 [−2.09, 1.57]	0.757	0.719
Hct, %^†^	38.8 (5.34)	38.9 (1.68)	40	0.14 [−4.19, 3.99]	0.660	0.549
MCV, fL^†^	85.5 (17.09)	91.6 (4.89)	37	0.06 [−19.2, 6.83]	0.884	0.791
MCH, pg^†^	27.5 (6.60)	30.0 (2.58)	30	−0.14 [−7.64, 2.64]	0.669	0.788
MCHC, g/dL^†^	31.9 (2.35)	32.7 (1.54)	25	−0.16 [−2.75, 1.22]	0.378	0.992
RDW, fL^†^	15.3 (3.47)	13.4 (3.51)	51	0.47 [−1.41, 5.33]	0.118	0.582
PLT, × 10^9^/mm^3^ ^†^	306.4 (136.06)	238.0 (56.34)	47	0.34 [−38.24, 175.1]	0.270	0.881
MPV, fL	8.6 (1.61)	9.0 (1.310)	−0.66	−0.33 [−1.91, 0.97]	0.518	0.276
Neutrophil, %	59.0 (9.52)	56.1 (8.92)	0.66	0.32 [−6.0, 11.92]	0.522	0.698
Lymphocyte, %	31.8 (7.00)	33.7 (8.22)	−0.50	−0.25 [−9.18, 5.36]	0.625	0.804
Monocyte, %	5.2 (1.19)	6.4 (1.90)	−1.58	−0.78 [−2.75, 0.19]	0.136	0.213
Eosinophil, %	2.4 (1.53)	2.6 (1.60)	35.5	0.01 [−1.71, 1.31]	1.000	0.828
Basophil, %	0.3 (0.53)	0.5 (0.32)	−1.32	−0.65 [−0.44, 0.07]	0.208	0.253
ANC, /µL	4519.4 (1810.14)	3045.3 (1302.59)	1.96	0.97 [−91.13, 3039.38]	0.069	0.539
CRP, mg/L^†^	2.73 (2.56)	0.15 (0.09)	47.5	0.47 [−0.42, 3.38]	0.092	0.279
HbA1c, %^†^	12.3 (2.86)	5.2 (0.32)	26.0	−0.26 [−1.17, 3.08]	1.000	0.425
IL‐6^†^	4.1 (4.08)	2.4 (0.87)	39.5	0.13 [−1.29, 4.85]	0.080	0.783

^†^ For parametric variables, Student's t‐test or Mann‐Whitney U^†^ test was performed, and for non‐parametric variables, the Fisher's exact test was performed. *, *p*<0.05; **, *p*<0.01, ***, *p*<0.001. Adjusted for covariates (age) using linear regression residuals for numeric variables only; significance tested via permutation (*n* = 500).

Abbreviations: ANC, absolute neutrophil count; BW, body weight; CRP, C‐reactive protein; CI, confidential interval; DBP, diastolic blood pressure; HAM‐ A, Hamilton anxiety rating scale; HAM‐D, Hamilton depression rating scale; Hb, hemoglobin; HbA1c, hemoglobin A1c; Hct, hematocrit; IL‐6; interleukin 6; MCH, mean corpuscular hemoglobin; MCHC, mean corpuscular hemoglobin concentration; MCV, mean corpuscular volume; MPV, mean platelet volume; OR, odd ratio; PLT, platelet; PR, pulse rate; RBC, red blood cell; RDW, red cell distribution width; SBP, systolic blood pressure; WBC, white blood cell.

The use of self‐report questionnaires indicated that subjective stress perception, measured via the Stress Visual Scale (p = 0.016), was higher in the patient group (62 points) than in the control group (33.3 points). Stress perception, as measured using the Perceived Stress Scale (PSS, *p* = 0.002), was also elevated in the patient group. Suicidal ideation was more prevalent in the patient group (*p* = 0.003). The severities of depressive symptoms (assessed using the Hamilton Depression Rating Scale, HAM‐D, p = 0.001) and anxiety symptoms (measured using the Hamilton Anxiety Rating Scale, HAM‐A, p = 0.001) were both higher in the patient group. Furthermore, participants in the patient group had higher scores on the IOR subscale of the Schizotypal Personality Questionnaire (SPQ) than those in the control group (*p* = 0.001). Traumatic events, assessed using LEC‐5 (Life Events Checklist), were more frequent in the patient group (4.29 vs. 1.2 events, 0.009). Following adjustment for age using linear regression residuals, significant differences between groups were observed in only four variables: PSS (F = 4.61, η^2^ = 0.24, p = 0.047), HAMA (F = 13.49, η^2^ = 0.47, p = 0.001), HAMD (F = 11.65, η^2^ = 0.44, p = 0.004), and SPQ‐IOR (F = 14.10, η^2^ = 0.48, p = 0.003).

Blood tests revealed that the patient group exhibited elevated WBC counts. There were no between‐group differences in neutrophil, monocyte differential counts, C‐reactive protein levels as well as IL‐6 levels. After adjusting for age, elevated WBC count was not significantly different between groups.

### 2.2 Plasma Protein Expression Changes in Patients with Atypical Depression and Psychotic Symptoms

**Figure 1:**
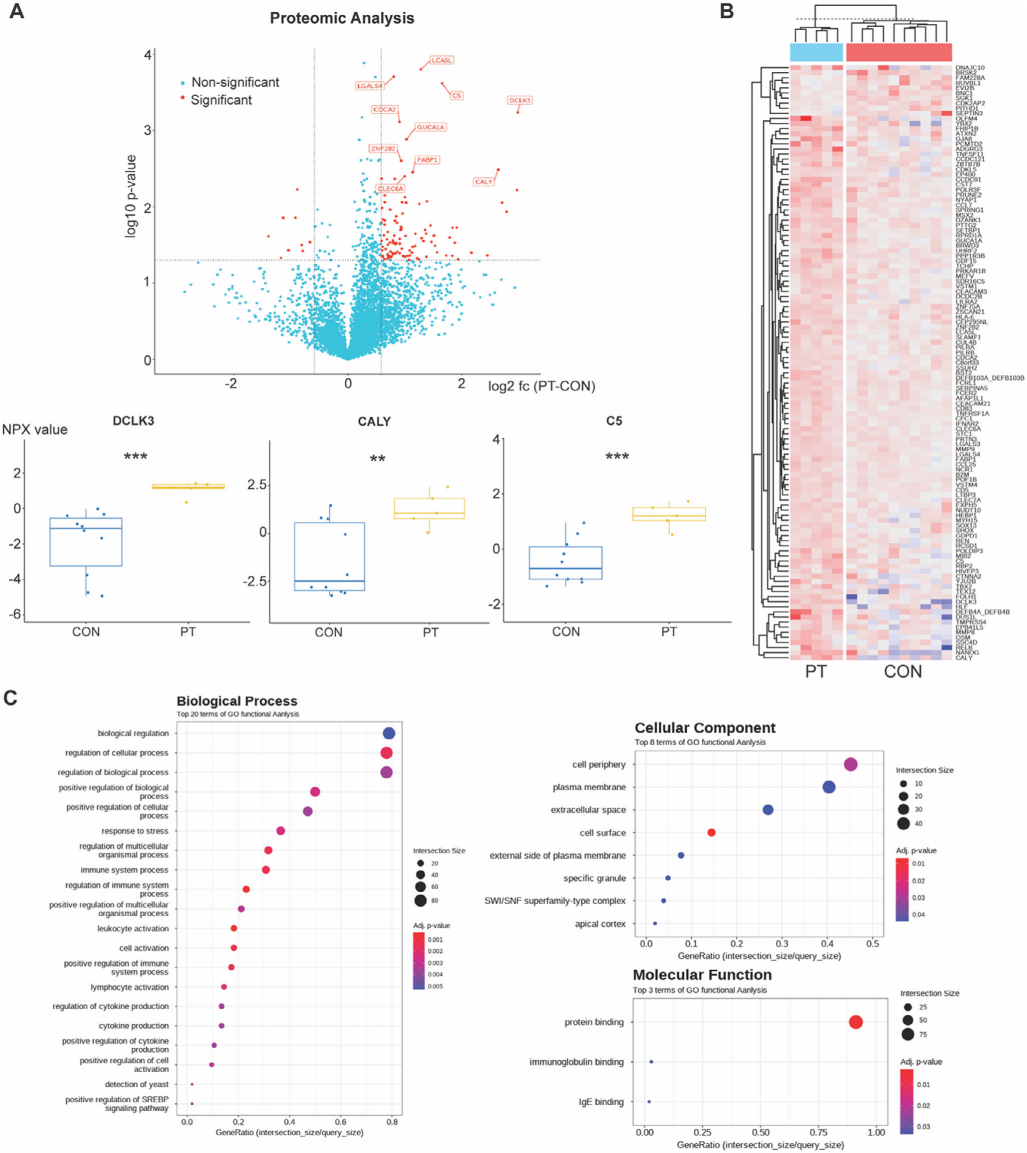
Plasma Proteomic Finding from Patients with Atypical Depression and Psychotic Symptoms. A) A volcano plot showed increased expression of plasma proteins including DCLK3, CALY, C5 in the patient group (PT) compared to the control group (CON). Box plots of normalized protein expression (NPX values) of DCLK3, CALY, and C5 are shown for PT (yellow) and CON (blue) groups. Compared with controls, PT samples exhibited significantly elevated levels of DCLK3 (***p < 0.001), CALY (**p < 0.01), and C5 (***p < 0.001). B) A heat map of the two‐way hierarchical clustering to differentiate plasma protein expression between PT and CON. C) Gene ontology (GO) term analyses showed the most prominent changes in regulating cellular process in the biological process, cell periphery in the cellular component, and protein binding in the molecular function.

Plasma proteomic analysis was performed on 5 patients and 10 healthy controls using proximity extension assay (**Figure** **1**). To explore overall proteomic differences, we performed principal component analysis (PCA), which revealed that the first principal component (PC1) accounted for 38.5% of the total variance and clearly separated the control and patient groups along the primary axis. This indicates a robust global difference in protein expression profiles between the two groups (Figure S1A, Supporting Information).

In proteomic results, the highest level of expression increase was observed in DCLK3 (Doublecortin Like Kinase 3) (log2 fold change, 2.99), a neuron‐specific kinase protein known for its role in neuronal survival and protection, ^[^ ^50^ ^]^ followed by NANOG (Homeobox Transcription Factor NANOG)^[^ ^51^ ^]^ (2.97), which are embryonic and neural stem cell‐related transcription factors, DUS1L (Dihydrouridine Synthase 1 Like) (2.79), and DEFB4A_DEFB4B (Defensin Beta 4A and 4B) (2.72). CALY (Calcyon Neuron‐Specific Vesicular Protein) (2.65), involved in nervous system functions such as synapse formation, ^[^ ^52^ ^]^ showed similarly high expression. Immune‐related proteins including OLFM4 (Olfactomedin 4) (2.45), RELB (RELB Proto‐Oncogene, NF‐κB Subunit) (2.18), C5 (Complement Component 5) (1.66), and SSC4D (Scavenger Receptor Cysteine Rich Family Member with 4 Domains) (1.94) also exhibited notable increases. Additionally, proteins such as DNAJC10 (DnaJ Heat Shock Protein Family Member C10) (1.91), YBX2 (Y‐Box Binding Protein 2) (1.84), HLF (Hepatic Leukemia Factor) (1.79), and PCMTD2 (Protein‐L‐Isoaspartate (D‐Aspartate) O‐Methyltransferase Domain Containing 2) (1.84), which are involved in various cellular functions including transcription regulation, protein folding and degradation, and RNA binding, showed more than 3‐fold increases in expression (Figure 1A,B and Table S1, Supporting Information). None of the proteins remained significant after global FDR correction; however, several of the same candidates demonstrated consistent group differences in our independent validation, supporting the plausibility of these signals.

Functional annotation analysis revealed that significantly regulated proteins were predominantly associated with molecular functions such as protein binding and immunoglobulin binding, cellular components including the plasma membrane and extracellular space, and biological processes related to immune responses, leukocyte activation, and cellular regulation (Figure 1C).

Correlation analyses revealed that DCLK3 expression was positively associated with the Visual Stress Scale (r = 0.607), SPQ‐IOR total score (r = 0.702), LEC‐5 event count (r = 0.567), PSS (r = 0.608), HAM‐D (r = 0.620), and HAM‐A (r = 0.652). CALY expression correlated with the SPQ‐IOR total score (r = 0.516), LEC‐5 event count (r = 0.549), HAM‐D (r = 0.620), and HAM‐A (r = 0.644). C5 showed strong correlations with the SPQ‐IOR total score (r = 0.779), LEC‐5 event count (r = 0.699), PSS (r = 0.467), HAM‐D (r = 0.591), and HAM‐A (r = 0.646). Remarkably, DUS1L expression exhibited the strongest correlation overall, with PSS (r = 0.812) (Figure S1B, Supporting Information)

To evaluate inflammation‐related responses to circulating factors, we conducted plasma treatment experiments using a commercially available human microglial cell line (Figure S1C,D, Supporting Information). Plasma samples from three patients (PT) with atypical depression and psychotic symptoms and three matched healthy controls (CON) were selected for this assay. Dexamethasone was applied for one week as a positive control to simulate microglial activation associated with chronic HPA axis stimulation in psychiatric disorders.^[^ ^48^ ^]^ Quantitative analysis of Iba1⁺ cell area revealed a significant increase in dexamethasone‐treated cells compared to vehicle‐treated controls. Notably, plasma from healthy controls did not induce a similar change, as the plasma CON group did not significantly differ from the vehicle or dexa groups. These findings suggest that circulating factors present in patient plasma may promote microglial activation, consistent with the elevated levels of immune‐related and neurodevelopmental proteins identified in the plasma proteomic analysis.

To validate the proteomics findings, we performed ELISA for selected proteins.

CALY levels were significantly higher in patients (61.10 ± 6.26 µg mL^−1^) compared to controls (44.01 ± 6.83; *p*  =  0.033), and DCLK3 levels also showed a significant increase in patients (12.17 ± 1.38) relative to controls (8.92 ± 1.01; *p*  =  0.034). In contrast, C5 levels did not differ significantly between patients (123.87 ± 22.90) and controls (87.51 ± 14.03; *p*  =  0.093). The lack of significance for C5 may be attributed to methodological differences between proteomics and ELISA—such as antibody specificity and assay sensitivity—or inter‐individual variability. Nonetheless, these ELISA results support the differential expression of neuron‐associated proteins, particularly CALY and DCLK3 (Figure S1E, Supporting Information).

### 2.3 WBC Single‐Cell Transcriptomic Alteration in Patients with Atypical Depression and Psychotic Symptoms

**Figure 2:**
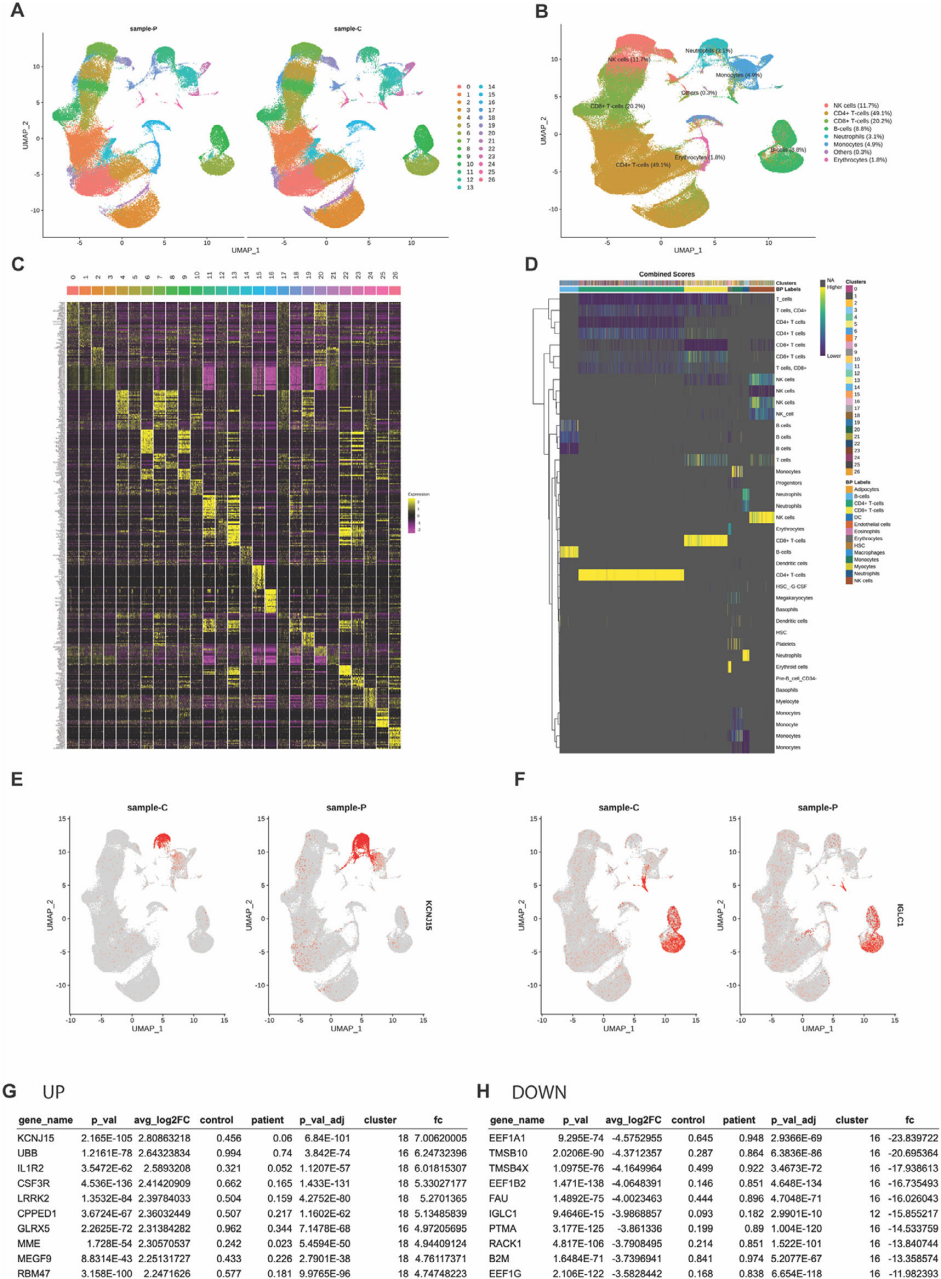
Differential WBC Single‐cell Transcriptomic Profiles in the Patient Group Elevated Inflammatory Gene Expression in Neutrophils and Monocytes. A–D) Identified cell types by Seurat analysis from single‐cell RNA sequencing in WBC between the patient (sample‐P) and healthy control (sample‐C) groups. Graph theory‐based cell type clustering of **B** is presented. Heatmap showing the gene expression patterns for the top genes in each cluster (C and D). E,F) FeaturePlot showing differentially expressed genes between the patient and healthy control groups at the cell level for each cluster. The expression level of *KCNJ15* increased more than seven times (fc 7.01) in neutrophils of the patient group compared with those of the healthy control group (E). The expression level of *IGLC1* decreased more than 15 times (fc ‐15.86) in B cells of the patient group compared with those of the healthy control group (F). G,H) List of the top 10 differentially expressed genes of single‐cell RNA sequencing of WBCs between the patient and healthy control groups.

In this study, WBC single‐cell RNA sequencing (scRNA‐seq) was performed on 6 patients and 10 healthy controls, with data analyzed using the Seurat package^[^ ^53^ ^]^ (Figure 2).

We observed significant differences in immune cell composition between the patient and control groups across multiple clusters (Table S2, Supporting Information). Specifically, clusters corresponding to CD4⁺ T cells (clusters 0–3), CD8⁺ T cells (clusters 4–6), B cells (clusters 10–12), and NK cells (clusters 7, 9) were markedly reduced in the patient group, whereas clusters corresponding to neutrophils (clusters 13–15), and monocytes (clusters 16–17) were significantly increased. These findings suggest a shift toward innate (myeloid cells) immune activation accompanied by a reduction in adaptive (lymphocytes) immune components in the patient group. The relative depletion of lymphocyte subsets, accompanied by an increase in myeloid‐derived cells, may reflect a pro‐inflammatory state and stress‐related immune remodeling.

The WBC scRNA‐seq results revealed notable transcriptomic differences between the groups. In cluster 18, classified as neutrophils, the patient group exhibited higher expressions of genes, such as *KCNJ15* (fold change, [fc] 7.01), *IL1R2* (fc 6.01), *CSF3R* (fc 5.33), *LRRK2* (fc 5.27), *CPPED1* (fc 5.13), *GLRX5* (fc 4.97), *MME* (fc 4.94), *MEGF9* (fc 4.76), and *RBM47* (fc 4.75).

In cluster 16, classified as monocytes, *UBB* (fc 6.24) was also highly expressed in the patient group. Conversely, in cluster 16, decreased transcriptomic expression was observed in the patient group for genes, such as *EEF1A1* (fc 23.84), *TMSB10* (fc 20.70), *TMSB4X* (fc 17.94), *EEF1B2* (fc 16.74), *FAU* (fc 16.03), *PTMA* (fc 14.53), *RACK1* (fc 13.84), *B2M* (fc 13.36), and *EEF1G* (fc 11.98).

In cluster 12, classified as B cells, *IGLC1* (fc 15.86) showed reduced expression in the patient group.

### 2.4 Growth and Single‐Cell Transcriptomic Changes in Brain Organoids in a Patient with Atypical Depression and Psychotic Symptoms

**Figure 3:**
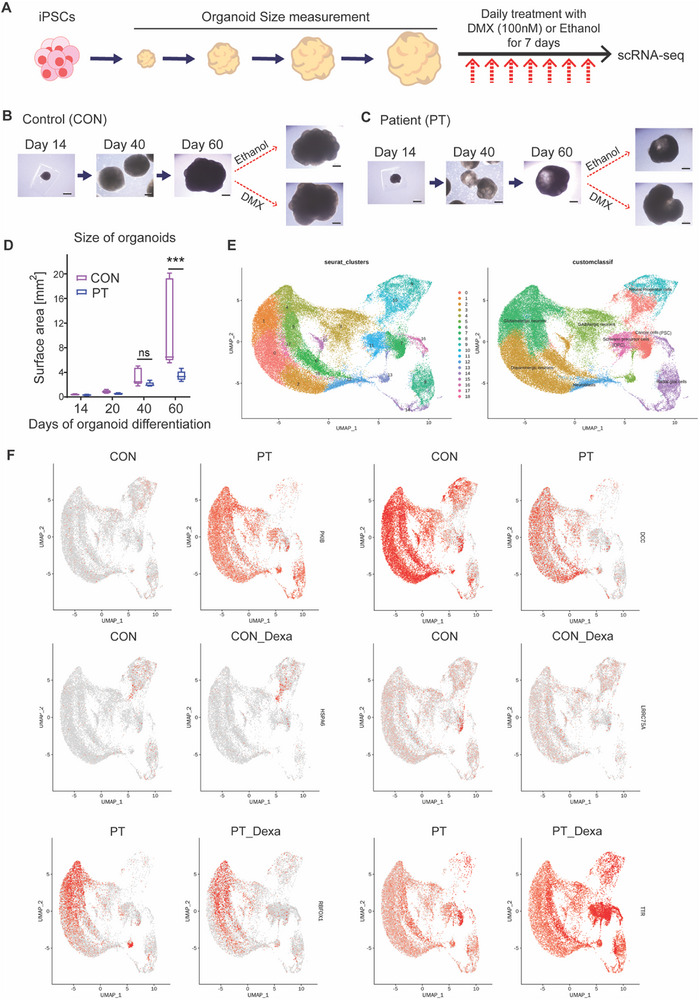
Transcriptomic Alterations in Brain Organoids Derived from a Patient with Atypical Depression and Psychotic Symptoms Increased Variability in Response to Dexamethasone Treatment. A) Experimental scheme for single‐cell RNA sequencing. Brain organoids were generated using induced pluripotent stem cells (iPSCs) derived from both a patient (PT) and a healthy control (CON). Organoids were cultured for 60 days. To simulate stress conditions, the organoids were treated with dexamethasone (DMX, 100 nM) or ethanol for 1 week. B) Bright‐field images showing CON brain organoids on days 14, 40, and 60, as well as after treatment with DMX or ethanol. Scale bar = 500 µm. C) Bright‐field images showing PT organoids on days 14, 40, and 60, as well as after treatment with DMX or ethanol. Scale bar = 500 µm. D) The brain organoids derived from the patient group were smaller than those from the healthy control group on day 60. Student's t‐test (*n* = 5 CON, *n* = 5 PT) ***, *p*<0.001; ns, not significant. E) Cell types identified by Seurat analysis of single‐cell RNA sequencing of brain organoids. Graph theory‐based cell type clustering is presented. F) FeaturePlot showing differentially expressed genes between patients and healthy controls at the cellular level for each cluster. In the patient group, decreased expression of *DCC* was observed in clusters 13 (GABAergic neurons), 17 (glutamatergic neurons), 5 (glutamatergic neurons), 18 (neuroblasts), and 1 (glutamatergic neurons). In contrast, increased expression of *PKIB* was observed in clusters 4 (glutamatergic neurons), 15 (dopaminergic neurons), 5 (glutamatergic neurons), 1 (glutamatergic neurons), 8 (radial glial cells), 3 (GABAergic neurons), and 17 (glutamatergic neurons). When comparing the CON and CON_Dexa groups, *LRRC75A* expression was reduced, whereas *HSPA6* expression was increased in cluster 10 (cancer cells) in the CON_Dexa group. In the PT and PT_Dexa groups, *TTR* expression increased in clusters 10 (cancer cells), 13 (GABAergic neurons), 7 (cancer cells), 11 (Schwann precursor cells), 8 (radial glial cells), 16 (radial glial cells), 14 (radial glial cells), and 15 (dopaminergic cells), whereas decreased expression was observed for *RBFOX1* in cluster 13 (GABAergic cells).

To investigate changes in patient brain organoids, monocytes from a patient were reprogrammed into iPSCs using the Sendai virus and subsequently differentiated into brain organoids. As a control, brain organoids were generated from iPSCs (obtained from adipose tissue‐derived mesenchymal stem cells from a healthy female) provided by the National Stem Cell Bank in South Korea (**Figure** **3**; Figure S2, Supporting Information).^[^ ^54^ ^]^ These organoids were cultured for 60 days, followed by a 1‐week treatment with DMX, a potent corticosteroid, to simulate stress‐induced HPA axis activation (Figure 3A). A size difference in the brain organoids between the patient and control groups became apparent, particularly on day 60 (Figure 3B).

The observed reduction in brain organoid size by day 60 in the patient group appears to reflect a fundamental impairment in early neurodevelopmental processes. Compositional analyses of brain organoid scRNA‐seq data revealed a significant depletion of neural progenitor cells (Cluster 9), alongside a shift toward glial‐like populations (Clusters 8 [radial glial cells], and 18 [neuroblast]), potentially indicating lineage misspecification or premature differentiation (Table S4, Supporting Information).

scRNA‐seq results for the three organoids from each group revealed significant transcriptomic differences (Figure S2 and Table S5, Supporting Information). In the patient group, decreased expression was observed in genes such as *DCC* (clusters 13 [GABAergic neurons], 17 [glutamatergic neurons], 5 [glutamatergic neurons], 18 [neuroblast], and 1 [glutamatergic neurons]), *PMCH* (cluster 9 [neural progenitor cells]), *HTR2C* (cluster 7 [cancer cell]), and *TTR* (cluster 7 [cancer cell]), and *DPP10* (cluster 4 [glutamatergic neurons]). The following genes were highly expressed: *PKIB* (clusters 4 [glutamatergic neurons], 15 [dopaminergic neurons], 5 [glutamatergic neurons], 1 [glutamatergic neurons], 8 [radial glial cells], 3 [GABAergic neurons], and 17 [glutamatergic neurons]), *KCNMB2* (cluster 13 [GABAergic neurons]), *TSHZ2* (cluster 13 [GABAergic neurons]), and *CSMD1* (cluster 4 [glutamatergic neurons]).

When comparing brain organoids of controls before (CON) and after (CON_Dexa) DMX treatment, *LRRC75A* expression was reduced by fc 2.70 in the CON_Dexa group, while *HSPA6* (cluster 10 [cancer cells]) and *PMCH* (cluster 9 [Schwann precursor cells]) expressions increased by fc 2.24 and fc 2.23, respectively.

An analysis of brain organoids of patients without (PT) and with (PT_Dexa) DMX treatment (Table S6, Supporting Information) demonstrated increased expressions of *TTR* (clusters 10 [cancer cells], 13 [GABAergic neurons], 7 [cancer cells], 11 [Schwann precursor cells], 8 [radial glial cells], 16 [radial glial cells], 14 [radial glial cells], and 15 [dopaminergic cells]), *PMCH* (cluster 9 [neural precursor cells]), and *CHL1* (cluster 15 [dopaminergic cells]), with decreased expressions of *RBFOX1* (cluster 13 [GABAergic cells]), *NMB* (cluster 9 [neural precursor cells]), *LRRC75A* (cluster 7 [cancer cells]), *PTPRD, ANKS1B, KCNH7, KAZN, CCSER1, DLGAP1*, and *IGFBP5* (cluster 10 [cancer cells]).

Compared with the CON group, which showed two increased and one decreased transcriptomic changes (Table S3, Supporting Information), the PT group exhibited a greater response to DMX treatment, with 23 increased and 34 decreased transcriptomic changes, suggesting a more significant transcriptomic variability in the PT_Dexa group than in the CON_Dexa group.

Based on observations from plasma proteomics, single‐cell RNA sequencing of patient‐derived brain organoids revealed a significant downregulation of *CALY*, with fold changes of −1.12 and −1.13 in clusters 1 and 4, respectively. *DCLK3* expression was also reduced in cluster 2 (fc −1.09). Notably, following dexamethasone treatment, *DCLK3* expression increased (fc 1.09), suggesting a potential stress‐responsive regulatory mechanism.

To validate the organoid findings, we performed immunocytochemistry (ICC) and qPCR (Figure S4, Supporting Information). At day 67, ICC revealed a decrease in SOX2⁺ neural progenitors and TUJ1⁺ neurons in PT organoids, with PT organoids exhibiting a markedly higher proportion of cleaved caspase‐3⁺ (CC3⁺) cells (***p < 0.001), indicating enhanced apoptotic activity that may underlie impaired neural survival and differentiation.^[^ ^55^ ^]^ qPCR validated scRNA‐seq results, confirming upregulation of *PKIB* and *CSMD1* and downregulation of *DCC, HTR2C, TTR*, and *DPP10* in PT organoids.

Analyses using PBMC‐derived control iPSCs (CON‐PBMC) and an additional patient iPSC clone (PT‐clone‐A) further supported these findings (Figure S5, Supporting Information), corroborating the findings obtained from mesenchymal stem cell–derived control iPSCs. Bright‐field imaging and surface area quantification revealed that PT‐clone‐A organoids were significantly smaller than CON‐PBMC organoids from day 30. At day 40, ICC revealed reduced proportions of SOX2⁺ neural progenitors and TUJ1⁺ neurons together with an increased proportion of CC3⁺ apoptotic cells in PT‐clone‐A organoids compared with CON‐PBMC organoids (****p < 0.0001). qPCR further validated the transcriptomic alterations, showing increased *PKIB* expression and decreased *DCC, HTR2C, TTR*, and *DPP10* expression in PT‐clone‐A organoids, with *CSMD1* exhibiting an upward trend without statistical significance.

## 3 Discussion

In this study, we applied a multi‐omics approach that, to our knowledge, is the first to combine plasma proteomics, WBC single‐cell RNA sequencing, and patient‐derived brain organoids, together with clinical and laboratory assessments, to investigate MDD with atypical features accompanied by psychotic symptoms. This integrative framework enabled us to explore molecular signatures across immune and neural systems within the same individuals. Our findings point to dysregulation in both immune and neuronal pathways, as well as potential links to stress vulnerability, offering preliminary insights into the complex biology underlying this severe and under characterized MDD subtype.

Patients exhibited elevated levels of trauma exposure, anxiety, and depression, consistent with previous findings that individuals with serious mental illness (SMI) report higher rates of lifetime trauma compared to the general population.^[^ ^56^ ^]^ Such exposures are well‐documented to influence inflammatory processes, immune cell phenotypes, and psychiatric symptomatology.^[^ ^57^, ^58^, ^59^, ^60^, ^61^, ^62^, ^63^ ^]^ Our multi‐omic results further support the notion that traumatic experiences may leave lasting molecular traces across the immune and neural systems.

Plasma proteomics revealed significant alterations in proteins involved in neuronal signaling, immune regulation, and complement activation. Notably, DCLK3, CALY, and C5 emerged as key candidates. DCLK3, a neuron‐specific kinase predominantly expressed in the striatum and hippocampus, has been implicated in neuronal survival and stress responses.^[^ ^64^ ^]^ Previous studies have linked DCLK3 to neuroprotection in Huntington's disease and behavioral changes in knockout mice, suggesting a role in circuits relevant to psychiatric symptoms.^[^ ^65^ ^]^ Moreover, transcriptomic analyses have associated DCLK3 with schizophrenia, bipolar disorder, and MDD, indicating its involvement in shared molecular pathways and pleiotropic effect across major psychiatric illnesses.^[^ ^66^ ^]^ In our study, DCLK3 was upregulated in patient plasma and showed dynamic expression in patient‐derived brain organoids following dexamethasone exposure, indicating a potential compensatory response to cellular stress. Given its enrichment in stress‐sensitive regions and involvement in major psychiatric disorders, altered DCLK3 expression may reflect adaptive mechanisms to immune‐neural disruption and elevated allostatic load in MDD. CALY, a neuron‐enriched transmembrane protein involved in clathrin‐mediated endocytosis and dopaminergic signaling, regulates AMPA receptor internalization, axonal trafficking, and D1 receptor dynamics^[^ ^52^, ^67^, ^68^, ^69^, ^70^ ^]^ Dysregulation of CALY has been implicated in neuropsychiatric conditions such as ADHD and schizophrenia.^[^ ^71^, ^72^ ^]^ In our study, CALY was upregulated in plasma but downregulated in patient‐derived brain organoids, suggesting compartment‐specific alterations in synaptic and dopaminergic signaling. These opposing patterns between peripheral and central compartments may reflect distinct regulatory mechanisms or compensatory responses relevant to MDD pathophysiology.

C5, a terminal effector of the complement cascade, promotes neuroinflammation through its cleavage product C5a, which activates pro‐inflammatory signaling via the C5aR1 receptor.^[^ ^73^ ^]^ C5 has been implicated in neuronal injury, glutamatergic dysfunction, and cognitive impairment, with elevated levels reported in the cerebrospinal fluid of patients with schizophrenia and MDD.^[^ ^74^, ^75^, ^76^, ^77^, ^78^ ^]^ In our study, C5 was elevated in plasma, supporting immune activation as a potential contributor to MDD pathophysiology, particularly through mechanisms involving cortical thinning and synaptic dysregulation.

The WBC scRNA‐seq results were particularly notable in neutrophils and monocytes, with the latter having a potential central nervous system involvement. Genes such as *IL1R2* ^[^ ^79^ ^]^ and *Csf3R*, ^[^ ^80^ ^]^ which are key molecules in the inflammatory response, were highly expressed in neutrophils, suggesting elevated inflammation in the patient group. Additionally, *LRRK2* associated with Parkinson's disease, was upregulated and is also known to regulate microglial inflammation.^[^ ^81^ ^]^ *Ubb* ^[^ ^82^ ^]^ was highly expressed in monocytes and played a crucial role in antigen presentation and immune regulation. The downregulation of *B2m* ^[^ ^83^ ^]^ in neutrophils, which is involved in MHC class I, suggests alterations in immune signaling. Previous studies on MDD have shown limited findings in WBC scRNA‐seq. One study reported increased inflammatory and immune‐metabolic pathways in monocytes of patients with high CRP levels. ^[^ ^84^ ^]^ Although our study did not detect an increase in MMP8 levels, as seen in stress‐vulnerable mice, ^[^ ^85^ ^]^ our results align with those of existing studies on immune dysregulation in MDD.

In brain organoid experiments, patient blood‐derived organoids showed reduced growth by day 60 compared with those of healthy controls. Transcriptomic analysis revealed changes from immature neural stem cells to mature neurons. Key genes, such as *DCC* (neural migration and axon guidance^[^ ^86^ ^]^), *PMCH* (melanin‐concentrating hormone^[^ ^87^ ^]^), and *HTR2C* (serotonin 2C receptor associated with stress response^[^ ^88^ ^]^), were differentially expressed, highlighting disruptions in neural development and signaling. Following DMX treatment, genes involved in the cellular stress response, such as *LRRC75A* ^[^ ^89^ ^]^ and *HSPA6*, ^[^ ^90^ ^]^ showed altered expression in the controls, whereas the patient group exhibited increased expressions of *TTR*, ^[^ ^91^ ^]^ *PMCH, and CHL1*, ^[^ ^92^ ^]^ which are all associated with brain development. In comparison with the findings of Cruceanu et al., ^[^ ^48^ ^]^ our study utilized DMX to replicate HPA‐axis activation and observed greater transcriptomic changes in the patient group, indicating heightened brain vulnerability. Given the scarcity of brain organoid research in MDD, our study offers a pioneering approach that combines WBC scRNA‐seq and brain organoid models to explore personalized medicine for psychiatric disorders.

The consistent alterations in DCLK3 and CALY across plasma, WBC, and brain organoids support their potential as trans‐compartmental biomarkers of neural stress vulnerability. Notably, plasma proteomic profiles more closely mirrored changes in brain organoid scRNA‐seq than WBC data, suggesting that circulating proteins may more accurately reflect central nervous system alterations in this context. This finding highlights the value of integrating peripheral and neural readouts to identify clinically relevant biomarkers for neuropsychiatric disorders.

Several limitations must be acknowledged. The study included a small, all‐female cohort and focused exclusively on patients with MDD with atypical features and psychotic symptoms. While this design improves clinical homogeneity and may enhance the sensitivity for detecting biological signals, it limits generalizability. Future studies should include male participants and a broader range of MDD subtypes to validate and extend these findings. Moreover, brain organoid experiments were conducted using cells from a single patient and two controls with three technical replicates each. Although this design reduces technical variability and strengthens within‐subject consistency, it limits biological reproducibility. The observed transcriptomic changes may reflect individual‐specific features rather than universally relevant pathophysiological mechanisms. Incorporating multiple biologically independent donors will be critical in future work to establish the robustness and generalizability of identified molecular signatures. Likewise, although no proteins remained significant after global FDR correction, the nominal effects were biologically coherent and were partly recapitulated by independent ELISA results, making a purely stochastic explanation unlikely. These signals are therefore best regarded as hypothesis‐generating and will require replication in larger and age‐matched cohorts to determine their robustness and relevance. The lack of microglia in our brain organoid model is another notable limitation. As microglia originate from yolk sac–derived mesoderm, they are absent in ectoderm‐derived organoids. To address this, we used WBC scRNA‐seq to capture peripheral immune states and conducted microglial cell culture experiments using plasma. The findings suggest that circulating immune factors in MDD may influence microglial activity, underscoring the importance of studying neuroimmune interactions in a more physiologically relevant context.

Despite these limitations, our study demonstrates the feasibility and utility of combining multi‐omics data from the same individuals to investigate stress‐related immune–neural interactions in MDD. By focusing on a well‐defined clinical subgroup, we identified consistent biological alterations that may underlie heightened stress sensitivity. While some interpretations remain preliminary and associative, the convergence of plasma and organoid data strengthens the case for a biological link between peripheral inflammation and neural dysfunction.

Two directions appear particularly important for future validation and refinement. First, although the present study was not powered for independent molecular confirmation, external validation — ideally in age‐matched psychiatric cohorts and using public transcriptomic or proteomic resources — will be crucial to test whether alterations such as DCLK3, CALY, or complement dysregulation are reproducible in larger and independent samples. Second, advances in neuroimmune modeling — including microglia‐containing organoids and immune co‐culture systems using peripheral immune cells or microglia‐like cells derived from patient iPSCs — now permit more physiologically relevant interrogation of immune–neural crosstalk. Embedding such platforms in future work will be essential to determine whether the peripheral signals detected here exert causal effects on neural circuits in patient‐derived models, and to refine their translational relevance for precision psychiatry.

## 4 Conclusion

This study provides a pioneering approach by multi‐omics, combining WBC scRNA‐seq, plasma proteomics, brain organoid scRNA‐seq, and clinical data to explore the biological underpinnings of MDD with atypical features accompanied by psychotic symptoms. Our findings revealed immune and neuronal dysregulation – particularly in neutrophils, monocytes, and plasma proteins – and identified significant transcriptomic changes in brain organoids under stress‐mimicking conditions. These results suggest a heightened biological vulnerability to stress in this patient populations and emphasize the importance of immune‐neuronal interactions in the pathophysiology of MDD. Collectively, our work provides valuable insights for advancing precision medicine in psychiatry.

## 5 Experimental Section

### Participants

This study was approved by the Institutional Review Board of Inha University Hospital (2022‐08‐003). Korean female patients diagnosed with MDD with atypical features accompanied by psychotic symptoms such as IOR, were recruited as the patient group. Although participants were not age‐matched at recruitment, all were female and under the age of 43 with regular menstrual cycles, and age was included as a covariate in downstream analyses. Patients with psychotic symptoms may or may not meet criteria for psychotic features specifier of MDD. Psychiatric assessments for the patient group were conducted independently by two board‐certified psychiatrists, and diagnoses were determined based on DSM‐5 criteria. Healthy controls were recruited from among individuals without a psychiatric diagnosis, including those with mood disorders. Participants with neurological conditions, such as seizures, cerebrovascular disease, brain neoplasm, traumatic brain injury, or intellectual disabilities, were excluded from the study.

It was collected blood samples from the study participants, isolated white blood cells for single‐cell transcriptomic analysis, and performed plasma proteomics using the separated plasma. Furthermore, blood tests were performed to obtain a complete blood count and differential count and to measure the levels of inflammatory markers such as CRP and IL‐6. Among the participants enrolled in the study, one contributed only to the collection of clinical and laboratory data but did not provide samples for proteomics and WBC scRNA‐seq. Another participant provided a blood sample that was used for WBC scRNA‐seq and the generation of induced pluripotent stem cells (iPSCs) for brain organoid derivation. Given the limited volume of the sample and the priority placed on iPSC generation for downstream organoid modeling, proteomic profiling was not conducted in this case.

All individuals who agreed to participate underwent physical examination, which included measurements of height, weight, blood pressure, pulse, and body temperature, as well as the collection of psychiatric and medical histories. Self‐reported measures were used to assess mood (HAM‐D), anxiety (HAM‐A), ideas of reference (classified as a disturbance of thought content, are considered one of the psychotic symptoms, IOR subscale of the schizotypal personality questionnaires, SPQ‐IOR), perceived stress (visual scale and PSS), life events (LEC‐5), problematic drinking (the third question of Alcohol Use Disorders Identification Test‐Korean, AUDIT‐K), and suicidal ideation (the ninth question of Patient Health Questionnaire‐9, PHQ‐9). Additionally, the SPQ‐IOR subscale was selected over the PQ‐B (Prodromal Questionnaire – Brief version) and PDI (Peters et al. Delusions Inventory) due to its larger item pool and ability to capture trait‐like referential thinking, providing a dimensional assessment across clinical and non‐clinical groups in line with DSM‐5‐based diagnoses.

### Statistical Analyses

Statistical analyses were performed using R and Python (including scipy, statsmodels, and pandas) software. For parametric variables that followed a normal distribution, the Student's t‐test was applied, and two‐way ANOVA was used to evaluate the effects of multiple factors. The Mann‐Whitney U test was used for non‐normally distributed variables. The Fisher's exact test was performed for nonparametric variables. To adjust for the demographic factor of age, covariate adjustment was performed using linear regression residuals for numeric variables only; statistical significance was assessed using permutation testing (*n* = 500).^[^ ^49^ ^]^ To address the limitation of a small sample size, it was reported effect sizes (Cohen's *d* or Cliff's delta) and 95% confidence intervals for all group comparisons, as these provide a more robust basis for interpretation than post hoc power analyses in exploratory settings.

## Conflict of Interest

The authors declare no conflict of interest.

## Author Contributions

I.A. and S.C. contributed equally to this work. J.H. and Y.K. contributed to the conception and design of the study; SC and SHC contributed to the acquisition of clinical information and data; I.A. performed iPSC and brain organoid experiments; J.L. performed microglial cell experiments; I.A., S.C., J.H., and Y.K. wrote the manuscript and prepared the figures.

## Supporting information



Supporting Information

Supporting Information

## Data Availability

The data that support the findings of this study are available on request from the corresponding author. The data are not publicly available due to privacy or ethical restrictions.
