# Supplementary material for: Exploration of Novel Biomarkers Through a Precision Medicine Approach Using Multi‐Omics and Brain Organoids in Patients With Atypical Depression and Psychotic Symptoms
Source: Adv Sci (Weinh). 2025 Oct 31;13(4):e08383. doi: 10.1002/advs.202508383 (PMC12822433; doi:10.1002/advs.202508383)
Supplement: Supplementary file 1 — Supporting Information [file ADVS-13-e08383-s001.docx]

**Exploration of Novel Biomarkers Through a Precision Medicine Approach Utilizing Immunological Alterations and Brain Organoid in Patients with Atypical Depression and Psychotic Symptoms**

Insook Ahn ^1,#^, Soyeon Chang, MD ^2,#^, Jiyoung Lee ^2^, Seok-Ho Choi, MD ^2^, Jinju Han, Ph.D. ^1,*^, Yangsik Kim, MD, PhD ^2,*^

^1^ Graduate School of Medical Science and Engineering (GSMSE), Korea Advanced Institute of Science and Technology (KAIST), Daejeon, 34051, South Korea;

^2^ Department of Psychiatry, Inha University Hostpial, College of Medicine, Inha University, Incheon, 22332**,** South Korea.

^#^ Equal contribution

*Corresponding authors:

Jinju Han, Ph.D.

GSMSE, KAIST,

Daejeon, Republic of Korea

Tel: +82-42-350-0225

E-mail: jinjuhan@kaist.ac.kr

Yangsik Kim, MD, PhD.

Department of Psychiatry,

Inha University Hostpial

College of Medicine, Inha University,

Incheon, South Korea

Tel: +82-32-890-3493

E-mail: ykim@inha.ac.kr

**Supplementary methods and materials**

**Single cell RNA sequencing (scRNA-seq) – WBC and brain organoids**

Briefly, WBCs were isolated from whole blood of study participants, and dead cells were removed for WBC scRNA-seq. Five brain organoids per sample, differentiated for 67 days, were dissociated, and dead cells were removed for brain organoid scRNA-seq. cDNA libraries targeting 10,000 cells per sample were prepared using the 10x Chromium Single Cell 3’ Protocol (10x Genomics, CA, USA). The prepared libraries were sequenced on an Illumina HiSeq X system (Illumina, CA, USA).

For bioinformatic analysis, raw base call files were converted to FASTQ format using ‘mkfastq,’ and sorting, filtering, barcode counting, and UMI counting were performed with ‘cellranger.’ Subsequent analyses, including data preprocessing, quality control, dimensionality reduction, graph-based cell clustering, identification of top-expressed biomarkers, gene set enrichment (using gProfiler), differential gene expression between groups, cell subtype classification, and population analysis, were conducted using the Seurat 4.0.5 R package. Cell type classification was based on the method described by Zeisel et al. Single-cell RNA sequencing and bioinformatics analyses were performed by Macrogen (Seoul, South Korea). Mitochondrial, ribosomal, RBC-related and MALAT1 genes/isoforms were excluded from the analysis.

**Proteomic Analysis**

Proteomic profiling was performed using the Olink Explore HT platform based on Proximity Extension Assay (PEA) technology (Macrogen, South Korea). In brief, samples were incubated with paired antibodies conjugated to unique DNA oligonucleotides. Upon binding to target proteins, the oligonucleotides came into proximity, hybridized, and were extended by a DNA polymerase to form a unique DNA barcode. These barcodes were amplified by PCR, indexed, and pooled for library preparation. Following purification and quality control, libraries were sequenced on an Illumina® platform. A total of 5,416 protein assays were measured per sample. Protein abundance was quantified based on sequencing read counts, and Normalized Protein eXpression (NPX) values were calculated from raw counts using Olink’s Intensity normalization method.

To identify differentially expressed proteins (DEPs), statistical comparisons were conducted between groups. To control for false positives arising from multiple hypothesis testing, p-values were adjusted using the Benjamini-Hochberg false discovery rate (FDR) correction. Only proteins meeting both statistical significance thresholds (p-value < 0.05) and biological relevance criteria (e.g., fold-change thresholds) were considered as DEPs. This approach enhances the robustness and reproducibility of protein-level findings.

**Reprogramming of hiPSCs**

Four days prior to the introduction of reprogramming factors (referred to as day -4)**,** peripheral blood mononuclear cells (PBMCs) were collected according to the following procedure. A patient’s blood sample, collected in BD Vacutainer K2 EDTA (K2E) blood collection tubes, was diluted in 10 ml of DPBS (Welgene, LB201-02) and gently layered over 20 ml of lymphocyte separation medium (Corning 25-072-CV). The sample was centrifuged at 300×g for 20 minutes at room temperature (RT). After centrifugation, the middle layer containing PBMCs was carefully transferred to a 50 ml tube (SPL 50450). The cells were then washed twice with DPBS, followed by centrifugation at 300×g for 8 minutes each time at RT. Three million PBMCs were seeded into a 24-well plate (SPL 30024) at a density of 0.5 million cells per well and cultured in Stempro-34 medium (Gibco 10639-011) supplemented with 100 ng/ml SCF (Gibco PHC2111), 100 ng/ml FLT-3 (Gibco PHC9414), 20 ng/ml IL-3 (Gibco PHC0034), and 20 ng/ml IL-6 (Gibco PHC0065), referred to as the complete PBMC medium. The cells were cultured with half media changes every other day for 4 days.

On day 0, 0.5 million PBMCs were placed in a round-bottom tube and treated with Sendai viral particles using the CytoTune-iPS Sendai Reprogramming Kit (Gibco A16518), according to the manufacturer's instructions, at multiplicities of infection (MOIs) of KOS = 5, hc-Myc = 5, and hKlf4 = 3. The round-bottom tube containing the PBMC and virus mixture was centrifuged at 1000×g for 30 minutes at RT. After centrifugation, the PBMCs were resuspended in complete PBMC medium and transferred to a single well of a 12-well plate (SPL 30012). On day 1, cells were collected into a 15 ml tube (SPL 50215) and centrifuged at 200×g for 10 minutes at RT to remove Sendai viruses, then resuspended in complete PBMC medium in a 24-well plate and cultured for 2 days without media changes. On day 3, the reprogrammed cells were plated on mouse embryonic fibroblasts (MEFs), which had been inactivated with 10 μg/ml of mitomycin C (AG Scientific M-1108) for 2 hours. The Institutional Review Board of KAIST has approved the protocols for using MEFs in accordance with the relevant ethical standards and regulations (KH2021-069). From day 4 to day 6, half of the medium was changed with StemPro-34. On day 7, half of the medium was replaced with iPSC medium consisting of DMEM/F12 (Gibco 12400024) supplemented with 1% MEM non-essential amino acids (NEAA; Gibco 11140050), 14.3 mM sodium bicarbonate (Sigma S5761), 1 mM L-glutamine (Sigma S5792), 0.1 mM β-Mercaptoethanol (2-ME, Merck M3148), 20% knockout serum replacement (KSR, Gibco 10828028), and 10 ng/ml bFGF (R&D Systems 4114-TC). On day 8, a full media change with the same iPSC medium was performed. The reprogrammed iPSC colonies were monitored under a microscope until they emerged. The generated iPSCs were maintained on MEFs in iPSC medium and passaged onto new plates every 6 days using collagenase (Gibco 17104019).

**iPSC validation:** **Live cell imaging, Reverse transcription (RT)-PCR, Karyotyping**

TRA1-60 was visualized using the TRA-1-60 Alexa Fluor™ 488 Conjugate Kit for Live Cell Imaging (ThermoFisher A25618) according to the manufacturer’s instructions. Briefly, the molecular probe was centrifuged at 10,000×g for 2 minutes and then applied to the prepared iPSCs at a 1:50 ratio in culture media. The cells were incubated at 37°C for 30 minutes. After incubation, the cells were washed 2-3 times with FluoroBrite™ DMEM (ThermoFisher A1896701) and imaged using the Olympus IX71 microscope.

To validate the expression of genes associated with pluripotent stem cells, *KLF4*, *SOX2*, *OCT4* and *NANOG* were amplified by RT-PCR. Total RNA was first extracted under RNase-free conditions using TRIzol (Invitrogen 15596-018), chloroform (Junsei 28560-0350), isopropanol (Merck 109634.1011), and ethanol (Merck 100983.1011). cDNA was synthesized from the total RNA using oligo dT and the RevertAid First Strand cDNA synthesis kit (ThermoFisher K1621) according to the manufacturer’s instructions. PCR was then performed using the cDNA, Ex Taq (Takara R001A) and the following primers: KLF4_Fwd 5’ CGCTGGCGGGAGGAGCTCTC 3’, KLF4_Rev 5’ GGTGACAGTCCCTGCTGCTC 3’, SOX2_Fwd 5’ CCGTTCATCGACGAGGCTAA 3’, SOX2_Rev 5’ ATTGGTGTTCTCTTTTGCAG 3’, OCT4_Fwd 5’ CTTGGAGACCTCTCAGCCTG3’, OCT4_Rev 5’ AGCAGGGCTGGATGCCTTCA 3’, NANOG_Fwd 5’ GATACTCATAAAGCCGCTAC 3’, NANOG _Rev 5’ CCCCACTTGCTCATTCCCAA 3’, ACTB_Fwd 5’ CGAGCACGGCATCGTCACCAA 3’, ACTB _Rev 5’GCAGCACGGGGTGCTCCTCG 3’. PCR was performed using a Bio-RAD C-1000 Touch Thermal Cycler under the following conditions: denaturation at 95°C for 1 minute, annealing at 55°C to 65°C for 1 minute, and elongation at 72°C for 1 to 2 minutes, for a total of 30 amplification cycles.

Karyotyping analysis was performed by Dx&Vx, Inc. (Seoul, South Korea).

**Forebrain organoids differentiation**

We generated organoids following the protocol described by Qian et al. (Nature Protocols, 2018), using two control iPSC lines: one derived from female PBMCs provided by Cedars-Sinai (CS4NWCiCTR-nxx; CS4, kindly shared by Prof. Chunghun Lim at KAIST) and the other from female adipose tissue–derived mesenchymal stem cells obtained from the National Stem Cell Bank in South Korea, as well as two independent clones from a patient. In brief, on day 0, iPSCs were dissociated using collagenase and transferred to non-adherent dishes (SPL 10060) with media (1), containing 2 nM Dorsomorphin (STEMCELL Technologies 72102), 2 nM A-83 (STEMCELL Technologies 72022), 1% MEM NEAA, 0.1 mM 2-ME, 20% KSR, and 1% penicillin/streptomycin (PS) (Welgene LS202-02) in DMEM F-12/Glutamax (Gibco 10565042). A full media change was performed on day 1, and half media changes were done on days 3 and 4. On days 5 and 6, the organoids were transitioned to media (2), which contained 1 nM CHIR-99021 (STEMCELL Technologies 72052), 0.1 nM SB-431242 (Cayman CAY-13031), 1% MEM NEAA, 1% PS, and N2 supplement (Gibco 17502048) in DMEM F-12/Glutamax, with half media changes. On day 7, 6 organoids were embedded in 30 µl of Matrigel (Corning 354230) and incubated at 37°C, 5% CO2 for 40 minutes. The Matrigel-coated organoids were transferred to media (2) and cultured with half media changes every other day until day 13. On day 14, embedded organoids were dissected out of the Matrigel using a surgical scalpel (Jeungdo bio H-2201-3). The separated organoids were cultured on a shaker (N-BIOTEK 101SRC) rotating continously at 80 rpm in media (3), containing N2 and B27 supplement (Gibco12587010), 0.1 mM 2-ME, 1% PS, and 2.5 ng/ml insulin (Sigma I9278). Thereafter, the organoids were cultured with half media changes every other day. For the DMX (Sigma D4902) treatment, 100 nM DMX diluted in ethanol was added to the culture media daily for one week, starting on day 61 after the organoids were differentiated for 60 days. As a control, 100% ethanol was added to the organoids daily for one week, starting on day 61.

**Immunostaining of brain organoids**

Organoids were washed 1–2 times with Dulbecco’s phosphate-buffered saline (DPBS; Welgene, LB201-02) and fixed in cold 4% formaldehyde (Pierce, 28906) for 30–60 minutes at room temperature (RT). For cryoprotection, the fixed organoids were incubated in 30% sucrose solution at 4°C until they fully sank to the bottom of the conical tube. Organoids were then rinsed 2–3 times with DPBS and embedded in O.C.T. compound (Leica, 14020108926) using embedding molds (Sakura, 4566; Sungwon, K4556). The embedded samples were snap-frozen using dry ice and sectioned into 40-μm-thick slices using a cryostat (Leica CM3050S). Sections were mounted on glass slides (Marienfeld, HSU-0810001), and a hydrophobic barrier was drawn around the tissue using a hydrophobic barrier pen (Matsunami, HMA-GMP0010).

Sections were washed three times with DPBS, permeabilized, and blocked in 5% normal donkey serum (Abcam, ab7475) diluted in 0.1% Triton X-100 (Promega, H5141) in DPBS for 1 hour at RT. Samples were then incubated overnight at 4°C with primary antibodies diluted in blocking buffer. After a brief rinse followed by three washes in PBST (0.1% Tween-20 in DPBS), sections were incubated with fluorescent dye-conjugated secondary antibodies for 1 hour at RT. Following 5–6 additional washes in PBST, nuclei were counterstained with DAPI (Sigma, D9542) for 10 minutes at RT. Finally, sections were mounted with fluorescence mounting medium (DAKO, S3023) and imaged using a confocal microscope (Zeiss LSM980).

The following antibodies were used:
Primary antibodies – SOX2 (Cell Signaling Technology, 3579S), TUJ1 (Abcam, ab52623), Cleaved Caspase-3 (CC3; Cell Signaling Technology, 9661).
Secondary antibodies – Donkey anti-Rabbit IgG H&L (Alexa Fluor® 594; Abcam, ab150068), Donkey anti-Mouse IgG H&L (Alexa Fluor® 488; Abcam, ab150105).

For quantification of apoptotic cells, CC3-positive cells were counted from three regions of interest (ROIs) per organoid (n = 3 organoids per CON and PT). The proportion of CC3-positive cells was normalized to the total number of DAPI-stained nuclei using ImageJ software.

**Quantitative Real-Time PCR (qPCR)**

Total RNA was extracted from brain organoids using TRIzol Reagent (Invitrogen, 15596-018) according to the manufacturer’s instructions. Complementary DNA (cDNA) was synthesized from 1 μg of total RNA using oligo(dT) primers and the RevertAid First Strand cDNA Synthesis Kit (Thermo Fisher Scientific, K1621). Quantitative real-time PCR (qPCR) was performed using the SYBR Green PCR Master Mix (Applied Biosystems, 368702) on a real-time PCR system. Gene expression levels were quantified using the relative standard curve method and normalized to β-actin expression.

The following primer sequences were used:

PKIB_Fwd 5’- CCTCAAACTGGAGGCTCTCTCC-3’,

PKIB_Rev 5’- AGCACTCTTGATAGATTATGAGCC-3’,

CSMD1_Fwd 5’-CGAAGAGGTACAATCCTGTCCC-3’,

CSMD1_Rev 5’- CCAGTTCTGCTCCGTGGCAAAA-3’,

DCC_Fwd 5’- GGTGAGATGGAAACACTGGAGC-3’,

DCC_Rev 5’-CTGTCATGGCTGACACCTGGAA-3’.

HTR2C_Fwd 5’-GTGGACGCTTCAAATTCCCA-3’,

HTR2C_Rev 5’-CTTACTGCCATGATCACAAGG-3’,

TTR_Fwd 5’-CGTGCATGTGTTCAGAAAGGCTG-3’,

TTR_Rev 5’-CTCCTCAGTTGTGAGCCCATGC-3’,

DPP10_Fwd 5’-GGACCGAAACCAGTATGCTCTTC-3’,

DPP10_Rev 5’- CTGGAATCCACTTCCTCTGCCA-3’,

beta-actin_Fwd 5’- CGAGCACGGCATCGTCACCAA-3’

and beta-actin_Rev 5’-GCAGCACGGGGTGCTCCTCG-3’.

**Microglial Plasma Treatment and Immunocytochemistry**

HMC3 immortalized human microglia cell line was cultured in Eagle's minimum essential medium (EMEM; ATCC, VA) containing 10% fetal bovine serum (FBS). Cells were seeded onto glass coverslips in 24-well plates at a density of 1 × 10⁴ cells per well and cultured under standard conditions. The following day, the culture medium was replaced with medium containing 5% plasma obtained from either patients or healthy controls. As positive and negative controls, cells were treated with 1 μM dexamethasone (Sigma-Aldrich, MO) or vehicle, respectively, for 7 days to model chronic HPA axis activation.

After 7 days of treatment, cells were fixed with 4% paraformaldehyde (PFA) for 10 minutes at room temperature (RT), followed by three washes with 0.1% Triton X-100 in PBS (PBST) for 5 minutes each. Cells were then blocked with 4% normal goat serum (Vector Laboratories, CA) and 1% bovine serum albumin (BSA; Sigma-Aldrich, MO) in PBST for 1 hour at RT, and incubated overnight at 4 °C with a rabbit anti-Iba1 primary antibody (1:500; Wako, Japan) diluted in 1% BSA in PBST. The next day, cells were washed three times with PBST and incubated with an Alexa Fluor 488–conjugated goat anti-rabbit secondary antibody (1:500; Thermo Fisher Scientific, MA) in 1% BSA for 2 hours at RT. After additional PBST washes, nuclei were counterstained with DAPI (Vector Laboratories, CA), and coverslips were mounted using fluorescence mounting medium.

Confocal images were acquired at 20× magnification using a FLUOVIEW FV3000 confocal microscope (Olympus, Japan). Morphological activation was assessed by quantifying the area of Iba1⁺ cells using ImageJ software (NIH, MD).

**ELISA assay**

Quantification of protein levels was performed using sandwich ELISA kits according to the manufacturer’s instructions (MyBioSource, USA). The following human ELISA kits were used: DCLK3 (Human doublecortin-like kinase 3, MBS9325126, 96-strip), CALY (Human calcyon neuron-specific vesicular protein, MBS9342284, 96-strip), and C5 (Human Complement C5, MBS825091, 96-strip). Briefly, 50 μL of standards (S1–S6) or samples were added to the appropriate wells of the microplate, while blank wells received no reagent. Then, 100 μL of horseradish peroxidase (HRP)-conjugated reagent was added to each well except the blank wells. The plate was sealed with a closure membrane and incubated for 60 minutes at 37°C. After incubation, all wells were washed four times with the provided wash buffer. Subsequently, 50 μL each of Chromogen Solution A and Chromogen Solution B were added to each well. The plate was gently mixed and incubated in the dark for 15 minutes at 37°C. The reaction was terminated by adding 50 μL of Stop Solution. Optical density (OD) was measured at 450 nm using a microplate reader within 15 minutes of adding the Stop Solution.

**Supplementary Tables**

**Supplementary Table 1.** Detailed list of differentially expressed proteins of plasma proteomics between the patient group (PT) and healthy control group (CON), sorted by fold change (fc)

| **Protein** | **UniprotID** | **p_value** | **FDR** | **log2_FC (PT-CON)** |
| --- | --- | --- | --- | --- |
| DCLK3 | Q9C098 | 0.001 | 0.3920 | 2.990 |
| NANOG | Q9H9S0 | 0.006 | 0.9382 | 2.974 |
| DUS1L | Q6P1R4 | 0.012 | 0.9997 | 2.794 |
| DEFB4A_DEFB4B | O15263 | 0.009 | 0.9527 | 2.716 |
| CALY | Q9NYX4 | 0.003 | 0.8379 | 2.648 |
| OLFM4 | Q6UX06 | 0.043 | 0.9997 | 2.454 |
| RELB | Q01201 | 0.040 | 0.9997 | 2.176 |
| DNAJC10 | Q8IXB1 | 0.049 | 0.9997 | 1.940 |
| GJA8 | P48165 | 0.019 | 0.9997 | 1.914 |
| ADGRG3 | Q86Y34 | 0.030 | 0.9997 | 1.881 |
| YBX2 | Q9Y2T7 | 0.019 | 0.9997 | 1.843 |
| PCMTD2 | Q9NV79 | 0.044 | 0.9997 | 1.838 |
| HLF | Q16534 | 0.025 | 0.9997 | 1.791 |
| SSC4D | Q8WTU2 | 0.043 | 0.9997 | 1.729 |
| C5 | P01031 | 0.000 | 0.2468 | 1.660 |
| YJU2B | P13994 | 0.039 | 0.9997 | 1.573 |
| FOLH1 | Q04609 | 0.032 | 0.9997 | 1.534 |
| MIB2 | Q96AX9 | 0.024 | 0.9997 | 1.516 |
| TBX2 | Q13207 | 0.029 | 0.9997 | 1.473 |
| OSM | P13725 | 0.018 | 0.9997 | 1.453 |
| TNFSF11 | O14788 | 0.008 | 0.9527 | 1.392 |
| HIVEP3 | Q5T1R4 | 0.045 | 0.9997 | 1.385 |
| FHIP1B | Q8N612 | 0.022 | 0.9997 | 1.378 |
| CCDC91 | Q7Z6B0 | 0.020 | 0.9997 | 1.359 |
| MMP8 | P22894 | 0.028 | 0.9997 | 1.331 |
| CTNNA2 | P26232 | 0.026 | 0.9997 | 1.299 |
| LCA5L | O95447 | 0.000 | 0.2468 | 1.285 |
| RPRD1A | Q96P16 | 0.009 | 0.9527 | 1.259 |
| TEX12 | Q9BXU0 | 0.049 | 0.9997 | 1.249 |
| ZBTB7B | O15156 | 0.047 | 0.9997 | 1.220 |
| PPP1R3B | Q86XI6 | 0.023 | 0.9997 | 1.190 |
| POLR3F | Q9H1D9 | 0.033 | 0.9997 | 1.186 |
| FABP1 | P07148 | 0.004 | 0.8644 | 1.144 |
| EPB41L5 | Q9HCM4 | 0.033 | 0.9997 | 1.143 |
| CST7 | O76096 | 0.040 | 0.9997 | 1.128 |
| CCL7 | P80098 | 0.043 | 0.9997 | 1.098 |
| CEP295NL | Q96MC4 | 0.037 | 0.9997 | 1.088 |
| UHRF2 | Q96PU4 | 0.050 | 0.9997 | 1.078 |
| POLDIP3 | Q9BY77 | 0.044 | 0.9997 | 1.054 |
| GUCA1A | P43080 | 0.001 | 0.5973 | 1.032 |
| DZANK1 | Q9NVP4 | 0.009 | 0.9527 | 1.026 |
| GDF15 | Q99988 | 0.024 | 0.9997 | 1.017 |
| RBP2 | P50120 | 0.045 | 0.9997 | 1.011 |
| REN | P00797 | 0.007 | 0.9527 | 1.007 |
| CLEC6A | Q6EIG7 | 0.004 | 0.8644 | 1.003 |
| NYAP1 | Q6ZVC0 | 0.045 | 0.9997 | 1.002 |
| TMPRSS4 | Q9NRS4 | 0.044 | 0.9997 | 0.968 |
| PRUNE2 | Q8WUY3 | 0.012 | 0.9997 | 0.963 |
| FCER2 | P06734 | 0.009 | 0.9527 | 0.957 |
| BST2 | Q10589 | 0.036 | 0.9997 | 0.952 |
| CDKL5 | O76039 | 0.019 | 0.9997 | 0.942 |
| ZNF282 | Q9UDV7 | 0.002 | 0.6748 | 0.940 |
| GDPD1 | Q8N9F7 | 0.018 | 0.9997 | 0.937 |
| SDR16C5 | Q8N3Y7 | 0.020 | 0.9997 | 0.929 |
| DEFB103A_DEFB103B | P81534 | 0.030 | 0.9997 | 0.920 |
| DCDC2B | A2VCK2 | 0.026 | 0.9997 | 0.916 |
| SHOX | O15266 | 0.041 | 0.9997 | 0.915 |
| CDCA2 | Q69YH5 | 0.001 | 0.4172 | 0.911 |
| CEACAM3 | P40198 | 0.030 | 0.9997 | 0.910 |
| ATXN2 | Q99700 | 0.039 | 0.9997 | 0.871 |
| LILRA2 | Q8N149 | 0.016 | 0.9997 | 0.868 |
| RCSD1 | Q6JBY9 | 0.030 | 0.9997 | 0.854 |
| MEFV | O15553 | 0.013 | 0.9997 | 0.852 |
| TCHP | Q9BT92 | 0.031 | 0.9997 | 0.842 |
| SPRING1 | Q9H741 | 0.041 | 0.9997 | 0.834 |
| CD83 | Q01151 | 0.015 | 0.9997 | 0.830 |
| MMP9 | P14780 | 0.004 | 0.8644 | 0.829 |
| HEBP1 | Q9NRV9 | 0.024 | 0.9997 | 0.819 |
| LGALS4 | P56470 | 0.000 | 0.2468 | 0.808 |
| NUDT10 | Q8NFP7 | 0.029 | 0.9997 | 0.807 |
| PRKAR1B | P31321 | 0.030 | 0.9997 | 0.801 |
| EP400 | Q96L91 | 0.027 | 0.9997 | 0.794 |
| SOX13 | Q9UN79 | 0.029 | 0.9997 | 0.794 |
| MYH15 | Q9Y2K3 | 0.042 | 0.9997 | 0.793 |
| CCL25 | O15444 | 0.040 | 0.9997 | 0.786 |
| CEACAM21 | Q3KPI0 | 0.045 | 0.9997 | 0.766 |
| PTTG2 | Q9NZH5 | 0.039 | 0.9997 | 0.754 |
| LTBP3 | Q9NS15 | 0.011 | 0.9997 | 0.744 |
| EXPH5 | Q8NEV8 | 0.029 | 0.9997 | 0.742 |
| CUL4B | Q13620 | 0.046 | 0.9997 | 0.737 |
| ZSCAN21 | Q9Y5A6 | 0.044 | 0.9997 | 0.733 |
| CFC1 | P0CG37 | 0.045 | 0.9997 | 0.714 |
| SERPINA5 | P05154 | 0.006 | 0.9382 | 0.714 |
| CLEC7A | Q9BXN2 | 0.043 | 0.9997 | 0.708 |
| CCDC121 | Q6ZUS5 | 0.031 | 0.9997 | 0.705 |
| SLAMF1 | Q13291 | 0.021 | 0.9997 | 0.704 |
| IFNAR2 | P48551 | 0.042 | 0.9997 | 0.699 |
| NCR1 | O76036 | 0.043 | 0.9997 | 0.699 |
| MSX2 | P35548 | 0.048 | 0.9997 | 0.696 |
| VSTM4 | Q8IW00 | 0.027 | 0.9997 | 0.689 |
| PRTN3 | P24158 | 0.036 | 0.9997 | 0.665 |
| CD5 | P06127 | 0.009 | 0.9527 | 0.650 |
| PILRA | Q9UKJ1 | 0.007 | 0.9527 | 0.650 |
| LGALS3 | P17931 | 0.015 | 0.9997 | 0.647 |
| POF1B | Q8WVV4 | 0.017 | 0.9997 | 0.646 |
| VSTM1 | Q6UX27 | 0.049 | 0.9997 | 0.645 |
| SSUH2 | Q9Y2M2 | 0.024 | 0.9997 | 0.637 |
| B2M | P61769 | 0.006 | 0.9382 | 0.626 |
| HLA-E | P13747 | 0.045 | 0.9997 | 0.619 |
| FCRL1 | Q96LA6 | 0.015 | 0.9997 | 0.618 |
| AFAP1L1 | Q8TED9 | 0.033 | 0.9997 | 0.614 |
| STC1 | P52823 | 0.044 | 0.9997 | 0.612 |
| ZNF75A | Q96N20 | 0.044 | 0.9997 | 0.609 |
| TNFRSF1A | P19438 | 0.037 | 0.9997 | 0.606 |
| C8orf33 | Q9H7E9 | 0.010 | 0.9997 | 0.603 |
| PILRB | Q9UKJ0 | 0.004 | 0.8644 | 0.593 |
| SETBP1 | Q9Y6X0 | 0.028 | 0.9997 | 0.592 |
| BRWD3 | Q6RI45 | 0.016 | 0.9997 | 0.591 |
| SGK1 | O00141 | 0.029 | 0.9997 | -0.665 |
| PITHD1 | Q9GZP4 | 0.038 | 0.9997 | -0.790 |
| EVI2B | P34910 | 0.032 | 0.9997 | -0.810 |
| CDK2AP2 | O75956 | 0.006 | 0.9382 | -0.887 |
| BNC1 | Q01954 | 0.014 | 0.9997 | -0.921 |
| RUVBL1 | Q9Y265 | 0.037 | 0.9997 | -1.043 |
| FAM228A | Q86W67 | 0.014 | 0.9997 | -1.135 |
| BRSK2 | Q8IWQ3 | 0.047 | 0.9997 | -1.174 |
| SEPTIN3 | Q9UH03 | 0.024 | 0.9997 | -1.397 |

**Supplementary Table 2.** Differential Cell Cluster Abundance Between PT and CON Groups Based on WBC scRNA-seq data

| **Cluster** | **PT** |  | **CON** |  | **chi 2** | **p-value** |
| --- | --- | --- | --- | --- | --- | --- |
| **0** | 10938 | 14.9% | 13608 | 13.1% | 111.8 | <0.001 |
| **1** | 10681 | 14.5% | 12904 | 12.4% | 162.9 | <0.001 |
| **2** | 6654 | 9.0% | 11186 | 10.8% | 142.2 | <0.001 |
| **3** | 4054 | 5.5% | 12125 | 11.7% | 1975.4 | <0.001 |
| **4** | 3432 | 4.7% | 6059 | 5.8% | 116.3 | <0.001 |
| **5** | 3719 | 5.1% | 5515 | 5.3% | 5.7 | 0.017 |
| **6** | 3728 | 5.1% | 4875 | 4.7% | 12.8 | <0.001 |
| **7** | 4115 | 5.6% | 4266 | 4.1% | 210.5 | <0.001 |
| **8** | 3091 | 4.2% | 4608 | 4.4% | 5.8 | 0.016 |
| **9** | 2774 | 3.8% | 4663 | 4.5% | 55.5 | <0.001 |
| **10** | 2767 | 3.8% | 3755 | 3.6% | 2.5 | 0.114 |
| **11** | 3202 | 4.3% | 1342 | 1.3% | 1613.9 | <0.001 |
| **12** | 1262 | 1.7% | 3152 | 3.0% | 308.9 | <0.001 |
| **13** | 2147 | 2.9% | 2211 | 2.1% | 111.5 | <0.001 |
| **14** | 1889 | 2.6% | 2341 | 2.3% | 18.0 | <0.001 |
| **15** | 1337 | 1.8% | 1951 | 1.9% | 0.9 | 0.351 |
| **16** | 2547 | 3.5% | 154 | 0.1% | 3151.1 | <0.001 |
| **17** | 947 | 1.3% | 1372 | 1.3% | 0.4 | 0.546 |
| **18** | 1140 | 1.5% | 1155 | 1.1% | 64.1 | <0.001 |
| **19** | 977 | 1.3% | 1226 | 1.2% | 7.5 | 0.006 |
| **20** | 592 | 0.8% | 1580 | 1.5% | 182.5 | <0.001 |
| **21** | 432 | 0.6% | 1417 | 1.4% | 251.7 | <0.001 |
| **22** | 358 | 0.5% | 825 | 0.8% | 61.2 | <0.001 |
| **23** | 309 | 0.4% | 580 | 0.6% | 16.3 | <0.001 |
| **24** | 239 | 0.3% | 438 | 0.4% | 10.4 | 0.001 |
| **25** | 224 | 0.3% | 441 | 0.4% | 16.4 | <0.001 |
| **26** | 57 | 0.1% | 137 | 0.1% | 11.2 | <0.001 |

**Supplementary Table 3.** List of the top 20 differentially expressed genes of single-cell RNA sequencing of brain organoids between a patient and a healthy control, sorted by fold change (fc)

Down

| **gene_name** | **p_val** | **avg_log2FC** | **PT** | **CON** | **p_val_adj** | **cluster** | **fc** |
| --- | --- | --- | --- | --- | --- | --- | --- |
| DCC | 1.4369E-54 | -3.28 | 0.133 | 0.82 | 4.6132E-50 | 13 | -9.73 |
| PMCH | 2.2159E-16 | -2.96 | 0.173 | 0.475 | 7.1142E-12 | 9 | -7.79 |
| DCC | 8.0442E-33 | -2.63 | 0.228 | 0.918 | 2.5827E-28 | 17 | -6.20 |
| HTR2C | 1.0157E-21 | -2.50 | 0.004 | 0.164 | 3.2609E-17 | 7 | -5.66 |
| DCC | 2.604E-245 | -2.46 | 0.324 | 0.963 | 8.359E-241 | 5 | -5.51 |
| DCC | 5.2132E-31 | -2.45 | 0.635 | 1 | 1.6738E-26 | 18 | -5.48 |
| DCC | <2.225e-308 | -2.36 | 0.407 | 0.993 | <2.225e-308 | 1 | -5.14 |
| TTR | 2.6654E-40 | -2.36 | 0.581 | 0.824 | 8.5574E-36 | 7 | -5.13 |
| DCC | 7.413E-308 | -2.34 | 0.362 | 0.986 | 2.38E-303 | 4 | -5.07 |
| DPP10 | 1.465E-230 | -2.32 | 0.183 | 0.816 | 4.705E-226 | 4 | -4.99 |
| HTR2C | 6.5454E-19 | -2.30 | 0.087 | 0.582 | 2.1015E-14 | 10 | -4.92 |
| DCC | <2.225e-308 | -2.27 | 0.369 | 0.984 | <2.225e-308 | 0 | -4.82 |
| RSPO3 | 0.00027995 | -2.21 | 0.011 | 0.16 | 1 | 16 | -4.63 |
| UNC5D | 6.8663E-68 | -2.19 | 0.007 | 0.324 | 2.2045E-63 | 6 | -4.57 |
| DCC | 1.068E-168 | -2.19 | 0.335 | 0.936 | 3.427E-164 | 6 | -4.56 |
| DPP10 | 2.3542E-26 | -2.17 | 0.071 | 0.467 | 7.5585E-22 | 13 | -4.49 |
| TCF7L2 | 1.9103E-23 | -2.14 | 0.077 | 0.434 | 6.1334E-19 | 13 | -4.40 |
| UNC5D | 1.283E-252 | -2.07 | 0.014 | 0.546 | 4.119E-248 | 2 | -4.21 |
| DCC | <2.225e-308 | -2.06 | 0.373 | 0.99 | <2.225e-308 | 2 | -4.16 |
| AL157778.1 | 1.5951E-17 | -2.03 | 0.172 | 0.707 | 5.1212E-13 | 15 | -4.08 |

Up

| **gene_name** | **p_val** | **avg_log2FC** | **PT** | **CON** | **p_val_adj** | **cluster** | **fc** |
| --- | --- | --- | --- | --- | --- | --- | --- |
| AC069277.1 | 4.9481E-88 | 2.84 | 0.849 | 0.003 | 1.5886E-83 | 14 | 7.17 |
| AC069277.1 | 1.396E-259 | 2.84 | 0.795 | 0.01 | 4.483E-255 | 8 | 7.15 |
| PKIB | 5.671E-278 | 2.34 | 0.796 | 0.067 | 1.821E-273 | 4 | 5.08 |
| AC069277.1 | 1.266E-128 | 2.28 | 0.489 | 0.004 | 4.063E-124 | 6 | 4.86 |
| AC069277.1 | 5.511E-135 | 2.23 | 0.684 | 0.005 | 1.769E-130 | 12 | 4.69 |
| PKIB | 3.4662E-43 | 2.13 | 0.736 | 0.053 | 1.1129E-38 | 15 | 4.37 |
| PKIB | 6.421E-172 | 2.05 | 0.582 | 0.012 | 2.061E-167 | 5 | 4.15 |
| PKIB | <2.225e-308 | 2.04 | 0.809 | 0.033 | <2.225e-308 | 1 | 4.12 |
| KCNMB2 | 1.1361E-07 | 2.03 | 0.515 | 0.303 | 0.0036477 | 13 | 4.09 |
| HES5 | 0.00564366 | 2.01 | 0.269 | 0.111 | 1 | 16 | 4.04 |
| PKIB | 5.921E-183 | 2.01 | 0.701 | 0.07 | 1.901E-178 | 8 | 4.02 |
| PKIB | 6.017E-254 | 1.92 | 0.614 | 0.065 | 1.932E-249 | 3 | 3.79 |
| PKIB | 3.054E-21 | 1.92 | 0.691 | 0 | 9.8052E-17 | 17 | 3.79 |
| AC069277.1 | <2.225e-308 | 1.91 | 0.679 | 0.005 | <2.225e-308 | 2 | 3.77 |
| TSHZ2 | 1.2636E-15 | 1.91 | 0.638 | 0.279 | 4.057E-11 | 13 | 3.76 |
| CSMD1 | 1.0364E-76 | 1.89 | 0.521 | 0.19 | 3.3274E-72 | 4 | 3.70 |
| PKIB | 1.0726E-30 | 1.86 | 0.889 | 0.085 | 3.4437E-26 | 18 | 3.63 |
| PKIB | <2.225e-308 | 1.83 | 0.763 | 0.036 | <2.225e-308 | 0 | 3.57 |
| AC069277.1 | 1.7765E-18 | 1.83 | 0.459 | 0.016 | 5.7037E-14 | 13 | 3.55 |
| PKIB | 3.693E-91 | 1.81 | 0.417 | 0.027 | 1.1857E-86 | 6 | 3.49 |

**Supplementary Table 4.** Differential Cell Cluster Abundance Between PT and CON Groups Based on brain organoid scRNA-seq data

| **cluster** | **PT** |  | **CON** |  | **chi 2** | **p-value** |
| --- | --- | --- | --- | --- | --- | --- |
| **0** | 2582 | 17.1% | 2393 | 13.7% | 2.2 | 0.142 |
| **1** | 2076 | 13.8% | 1770 | 10.1% | 67.3 | <0.001 |
| **2** | 1685 | 11.2% | 1721 | 9.9% | 8.3 | 0.004 |
| **3** | 1221 | 8.1% | 1862 | 10.7% | 110.3 | <0.001 |
| **4** | 1463 | 9.7% | 1070 | 6.1% | 173.5 | <0.001 |
| **5** | 1049 | 7.0% | 1049 | 6.0% | 10.3 | 0.001 |
| **6** | 830 | 5.5% | 959 | 5.5% | 30.1 | <0.001 |
| **7** | 534 | 3.5% | 846 | 4.8% | 149.8 | <0.001 |
| **8** | 907 | 6.0% | 1019 | 5.8% | 286.8 | <0.001 |
| **9** | 185 | 1.2% | 1375 | 7.9% | 1840.1 | <0.001 |
| **10** | 103 | 0.7% | 1341 | 7.7% | 2256.0 | <0.001 |
| **11** | 621 | 4.1% | 451 | 2.6% | 835.2 | <0.001 |
| **12** | 570 | 3.8% | 645 | 3.7% | 2.3 | 0.128 |
| **13** | 392 | 2.6% | 122 | 0.7% | 918.9 | <0.001 |
| **14** | 317 | 2.1% | 292 | 1.7% | 49.2 | <0.001 |
| **15** | 87 | 0.6% | 283 | 1.6% | 203.0 | <0.001 |
| **16** | 93 | 0.6% | 81 | 0.5% | 463.9 | <0.001 |
| **17** | 246 | 1.6% | 73 | 0.4% | 109.4 | <0.001 |
| **18** | 126 | 0.8% | 94 | 0.5% | 27.4 | <0.001 |

**Supplementary Table 5.** List of the top 20 differentially expressed genes of single-cell RNA sequencing of brain organoids between a healthy control with or without dexamethasone treatment, sorted by fold change (fc)

Down

| **gene_name** | **p_val** | **avg_log2FC** | **PT_Dexa** | **PT** | **p_val_adj** | **cluster** | **fc** |
| --- | --- | --- | --- | --- | --- | --- | --- |
| AL589740.1 | 0.00018711 | -2.03 | 0.031 | 0.369 | 1 | 13 | -4.09 |
| PBX3 | 0.0001835 | -1.93 | 0.188 | 0.549 | 1 | 13 | -3.80 |
| ERBB4 | 7.8895E-05 | -1.93 | 0.375 | 0.689 | 1 | 13 | -3.80 |
| TENM3 | 0.01557005 | -1.91 | 0.312 | 0.508 | 1 | 13 | -3.75 |
| TCF7L2 | 0.00030282 | -1.87 | 0.094 | 0.434 | 1 | 13 | -3.65 |
| TAC1 | 0.00304307 | -1.77 | 0.125 | 0.393 | 1 | 13 | -3.41 |
| CRABP1 | 0.00889225 | -1.73 | 0.125 | 0.344 | 1 | 13 | -3.31 |
| AC096570.1 | 0.01809003 | -1.50 | 0.094 | 0.295 | 1 | 13 | -2.82 |
| LRRC75A | 9.4454E-17 | -1.43 | 0.037 | 0.163 | 3.0326E-12 | 7 | -2.70 |
| CNTN5 | 0.05928509 | -1.41 | 0.125 | 0.279 | 1 | 13 | -2.67 |
| HIST1H4C | 0.04458496 | -1.32 | 0.306 | 0.481 | 1 | 16 | -2.50 |
| NLGN1 | 0.00077345 | -1.27 | 0.061 | 0.321 | 1 | 16 | -2.41 |
| TTR | 0.03097761 | -1.24 | 0.719 | 0.918 | 1 | 13 | -2.36 |
| SSR2 | 8.329E-05 | -1.23 | 0.204 | 0.58 | 1 | 16 | -2.35 |
| EDIL3 | 0.00021438 | -1.22 | 0.125 | 0.484 | 1 | 13 | -2.33 |
| KLHL13 | 0.0019165 | -1.20 | 0.094 | 0.369 | 1 | 13 | -2.30 |
| SLC4A7 | 0.03323071 | -1.16 | 0.082 | 0.222 | 1 | 16 | -2.23 |
| WLS | 0.0003104 | -1.14 | 0.125 | 0.484 | 1 | 13 | -2.21 |
| UBE2C | 0.01298131 | -1.14 | 0.041 | 0.198 | 1 | 16 | -2.21 |
| HECW1 | 0.00167105 | -1.14 | 0.02 | 0.222 | 1 | 16 | -2.20 |

Up

| **gene_name** | **p_val** | **avg_log2FC** | **PT_Dexa** | **PT** | **p_val_adj** | **cluster** | **fc** |
| --- | --- | --- | --- | --- | --- | --- | --- |
| TTR | 0.0177317 | 2.59 | 0.8 | 0.932 | 1 | 14 | 6.03 |
| SPARCL1 | 0.22980245 | 2.00 | 0.102 | 0.049 | 1 | 16 | 4.00 |
| IGFBP5 | 0.09041268 | 1.59 | 0.245 | 0.148 | 1 | 16 | 3.02 |
| PRR16 | 0.00740844 | 1.55 | 0.188 | 0.049 | 1 | 13 | 2.93 |
| TEX14 | 0.25759235 | 1.38 | 0.062 | 0.148 | 1 | 13 | 2.60 |
| LMO4 | 0.89028444 | 1.31 | 0.25 | 0.287 | 1 | 13 | 2.48 |
| BCAN | 0.0334408 | 1.29 | 0.224 | 0.099 | 1 | 16 | 2.44 |
| AC012409.2 | 8.5944E-05 | 1.28 | 0.531 | 0.213 | 1 | 13 | 2.43 |
| SORCS3 | 0.03124673 | 1.28 | 0.375 | 0.221 | 1 | 13 | 2.42 |
| SYT11 | 0.08056664 | 1.27 | 0.306 | 0.21 | 1 | 16 | 2.40 |
| LINGO2 | 0.00029866 | 1.20 | 0.625 | 0.352 | 1 | 13 | 2.30 |
| HTR2C | 0.81049557 | 1.20 | 0.189 | 0.188 | 1 | 14 | 2.29 |
| GPC5 | 0.01946949 | 1.18 | 0.344 | 0.18 | 1 | 13 | 2.26 |
| ZFYVE16 | 0.00147781 | 1.17 | 0.286 | 0.086 | 1 | 16 | 2.25 |
| HSPA6 | 4.5903E-07 | 1.16 | 0.19 | 0.122 | 0.01473748 | 10 | 2.24 |
| PMCH | 3.0689E-26 | 1.16 | 0.648 | 0.475 | 9.853E-22 | 9 | 2.23 |
| LHFPL3 | 0.96996457 | 1.16 | 0.375 | 0.41 | 1 | 13 | 2.23 |
| RAB21 | 0.10368809 | 1.14 | 0.122 | 0.049 | 1 | 16 | 2.21 |
| ATCAY | 0.19920939 | 1.13 | 0.163 | 0.099 | 1 | 16 | 2.20 |
| SLIT2 | 0.0621193 | 1.13 | 0.406 | 0.279 | 1 | 13 | 2.19 |

**Supplementary Table 6.** List of the top 20 differentially expressed genes of single-cell RNA sequencing of brain organoids between a patient with or without dexamethasone treatment, sorted by fold change (fc)

Down

| **gene_name** | **p_val** | **avg_log2FC** | **CON_Dexa** | **CON** | **p_val_adj** | **cluster** | **fc** |
| --- | --- | --- | --- | --- | --- | --- | --- |
| RBFOX1 | 2.0127E-37 | -2.55 | 0.101 | 0.48 | 6.462E-33 | 13 | -5.85 |
| NFIA-AS2 | 1.6178E-37 | -2.00 | 0.043 | 0.221 | 5.1943E-33 | 7 | -3.99 |
| XIST | 2.2604E-35 | -1.71 | 0.007 | 0.175 | 7.2571E-31 | 6 | -3.28 |
| HES5 | 0.03833708 | -1.67 | 0.192 | 0.269 | 1 | 16 | -3.19 |
| XIST | 3.2075E-32 | -1.54 | 0.012 | 0.274 | 1.0298E-27 | 12 | -2.90 |
| AL157778.1 | 9.9533E-36 | -1.51 | 0.037 | 0.385 | 3.1956E-31 | 13 | -2.84 |
| XIST | 2.4125E-18 | -1.45 | 0.041 | 0.36 | 7.7457E-14 | 14 | -2.74 |
| NMB | 5.4988E-12 | -1.40 | 0.37 | 0.578 | 1.7655E-07 | 9 | -2.65 |
| XIST | 5.9374E-71 | -1.40 | 0.018 | 0.301 | 1.9063E-66 | 8 | -2.63 |
| XIST | 1.6072E-36 | -1.37 | 0.007 | 0.156 | 5.1602E-32 | 5 | -2.58 |
| LRRC75A | 8.8719E-20 | -1.34 | 0.032 | 0.137 | 2.8484E-15 | 7 | -2.54 |
| PTPRD | 2.4752E-26 | -1.31 | 0.141 | 0.337 | 7.947E-22 | 7 | -2.48 |
| ZFPM2-AS1 | 2.7089E-25 | -1.28 | 0.054 | 0.202 | 8.6973E-21 | 7 | -2.43 |
| LINC01456 | 2.3221E-39 | -1.27 | 0.108 | 0.523 | 7.4552E-35 | 13 | -2.41 |
| IL1RAPL1 | 5.9289E-06 | -1.24 | 0.128 | 0.291 | 0.19035313 | 10 | -2.37 |
| ANKS1B | 2.3116E-13 | -1.24 | 0.088 | 0.199 | 7.4215E-09 | 7 | -2.36 |
| KCNH7 | 5.6155E-17 | -1.24 | 0.109 | 0.249 | 1.8029E-12 | 7 | -2.35 |
| KAZN | 1.6176E-13 | -1.23 | 0.135 | 0.262 | 5.1934E-09 | 7 | -2.35 |
| FTX | 2.0207E-19 | -1.20 | 0.158 | 0.324 | 6.4878E-15 | 7 | -2.30 |
| CCSER1 | 8.7584E-21 | -1.19 | 0.135 | 0.305 | 2.812E-16 | 7 | -2.29 |

Up

| **gene_name** | **p_val** | **avg_log2FC** | **pct.1** | **pct.2** | **p_val_adj** | **cluster** | **fc** |
| --- | --- | --- | --- | --- | --- | --- | --- |
| TTR | 1.5336E-34 | 2.62 | 0.992 | 0.612 | 4.9238E-30 | 10 | 6.15 |
| TTR | 1.3393E-90 | 2.44 | 0.993 | 0.597 | 4.3001E-86 | 13 | 5.44 |
| PMCH | 5.4692E-08 | 2.37 | 0.372 | 0.173 | 0.00175595 | 9 | 5.17 |
| TTR | 4.747E-164 | 2.24 | 0.98 | 0.581 | 1.524E-159 | 7 | 4.71 |
| TTR | 3.197E-194 | 2.19 | 0.987 | 0.602 | 1.026E-189 | 11 | 4.57 |
| TTR | 3.263E-199 | 2.03 | 0.985 | 0.601 | 1.047E-194 | 8 | 4.10 |
| TTR | 1.1605E-30 | 2.03 | 0.993 | 0.516 | 3.726E-26 | 16 | 4.09 |
| TTR | 6.5753E-56 | 1.95 | 0.986 | 0.647 | 2.1111E-51 | 14 | 3.85 |
| CHL1 | 8.2962E-23 | 1.91 | 0.817 | 0.08 | 2.6636E-18 | 15 | 3.75 |
| TTR | 2.809E-24 | 1.82 | 1 | 0.621 | 9.0187E-20 | 15 | 3.53 |
| TTR | 2.686E-244 | 1.77 | 0.992 | 0.6 | 8.625E-240 | 3 | 3.40 |
| TTR | 2.142E-114 | 1.73 | 0.988 | 0.605 | 6.878E-110 | 12 | 3.31 |
| TTR | 2.159E-190 | 1.72 | 0.988 | 0.607 | 6.931E-186 | 5 | 3.30 |
| TTR | 4.502E-156 | 1.69 | 0.983 | 0.595 | 1.445E-151 | 6 | 3.23 |
| TTR | 2.6777E-40 | 1.67 | 1 | 0.589 | 8.5969E-36 | 17 | 3.18 |
| CCN2 | 5.8358E-07 | 1.62 | 0.291 | 0.058 | 0.01873649 | 10 | 3.08 |
| TTR | <2.225e-308 | 1.61 | 0.992 | 0.595 | <2.225e-308 | 2 | 3.06 |
| GULP1 | 1.6423E-13 | 1.60 | 0.581 | 0.194 | 5.2729E-09 | 10 | 3.04 |
| TTR | 6.58E-264 | 1.53 | 0.993 | 0.621 | 2.113E-259 | 4 | 2.89 |
| HTR2C | 1.6697E-09 | 1.47 | 0.41 | 0.087 | 5.3606E-05 | 10 | 2.78 |

**Supplementary Table 7. The full proteomic dataset**

The full proteomic dataset, including raw p-values and FDR-adjusted values for all identified proteins, is provided in Supplementary Table 7.

**Supplementary Figures and Figure Legends**

**
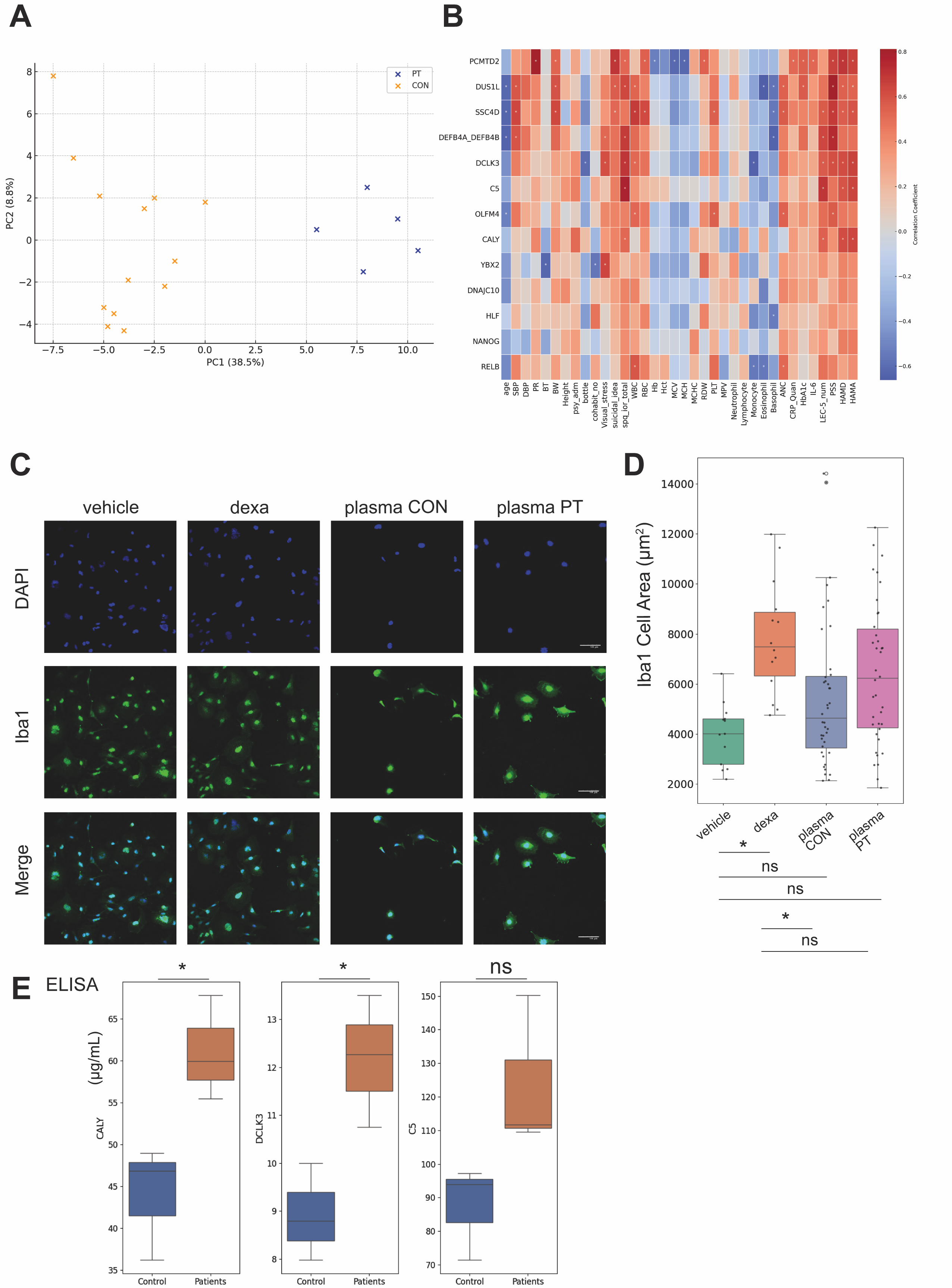
**

**Supplementary Figure 1. Multimodal profiling reveals distinct plasma signatures and microglial responses in patients with atypical depression accompanied with psychotic symptoms**

(A) Principal component analysis (PCA) revealed that PC1, accounting for 38.5% of the total variance, clearly separated PT and CON along the primary axis, suggesting a distinct global difference in protein expression profiles between the two groups.

(B) The heatmap displays correlation coefficients between significantly altered proteins identified through plasma proteomics and clinical variables. The color scale indicates the direction and magnitude of the correlation (red for positive, blue for negative), and asterisks denote statistical significance (p<0.05).

(C) Representative immunofluorescence images of microglia stained with Iba1 (green) and DAPI (blue) following treatment with vehicle, dexamethasone (dexa), control plasma (plasma CON), or patient plasma (plasma PT). Scale bar = 100 μm.

(D) Quantification of Iba1⁺ cell area (μm²) shows a significant increase in cell size in the dexa (n=14, mean 7826.6 μm², standard deviation 2271.89) and plasma PT (n=38 from 3 patients, 6404.9, 6234.184) groups compared to the vehicle (n=13, 3994.2, 1230.07) group. Notably, there was no significant difference between the dexa and plasma CON (n=40 from 3 controls, 5504.1, 2979.47) groups, suggesting that plasma from healthy controls does not elicit the same morphological activation in microglial cells as dexamethasone. Each dot represents an individual cell. Data are presented as boxplots with individual data points. Statistical comparisons were performed using Dunn’s post hoc test following Kruskal-Wallis analysis (*p < 0.05; ns = not significant).

(E) ELISA quantification (µg/mL) revealed significantly elevated levels of CALY and DCLK3 in patients (n=3) compared to controls (n=3), while C5 showed no significant difference. Statistical comparisons were performed using Student’s t-test (*p < 0.05; ns = not significant).


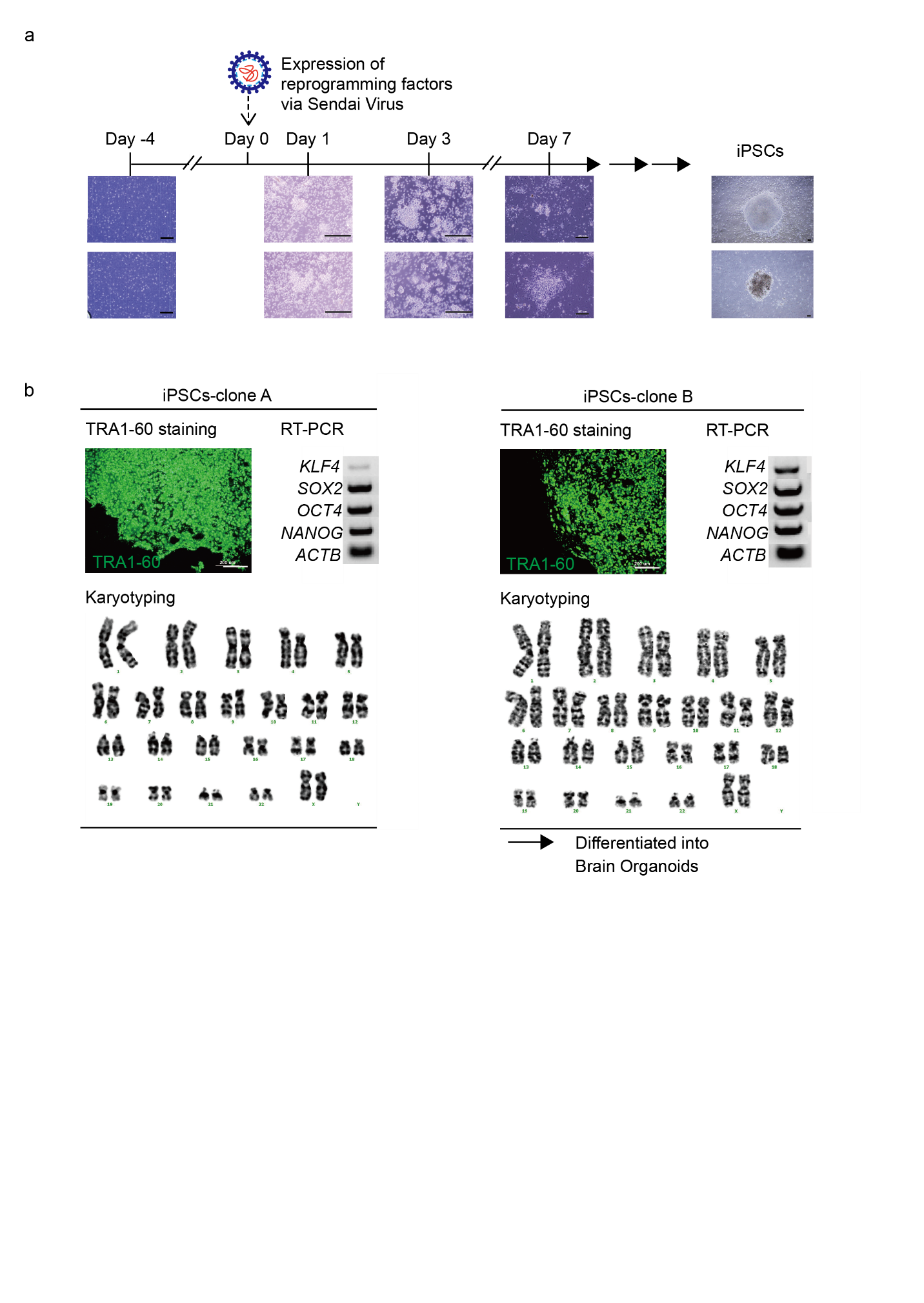


**Supplementary Figure 2. Generation and Characterization of iPSCs from PBMCs**

(A) Bright-field images showing the reprogramming process from PBMCs to the formation of iPSC colonies. Scale bar = 200μm.

(B) iPSC validation results. Live imaging of TRA1-60 staining of two iPSC colonies at passage 2. Scale bar = 200μm. Pluripotency marker expression (*KLF4*, *SOX2*, *OCT4*, and *NANOG*) was confirmed by RT-PCR, along with normal karyotype analysis, at passage 10. Among the two clones, clone B was used for organoid differentiation.

**
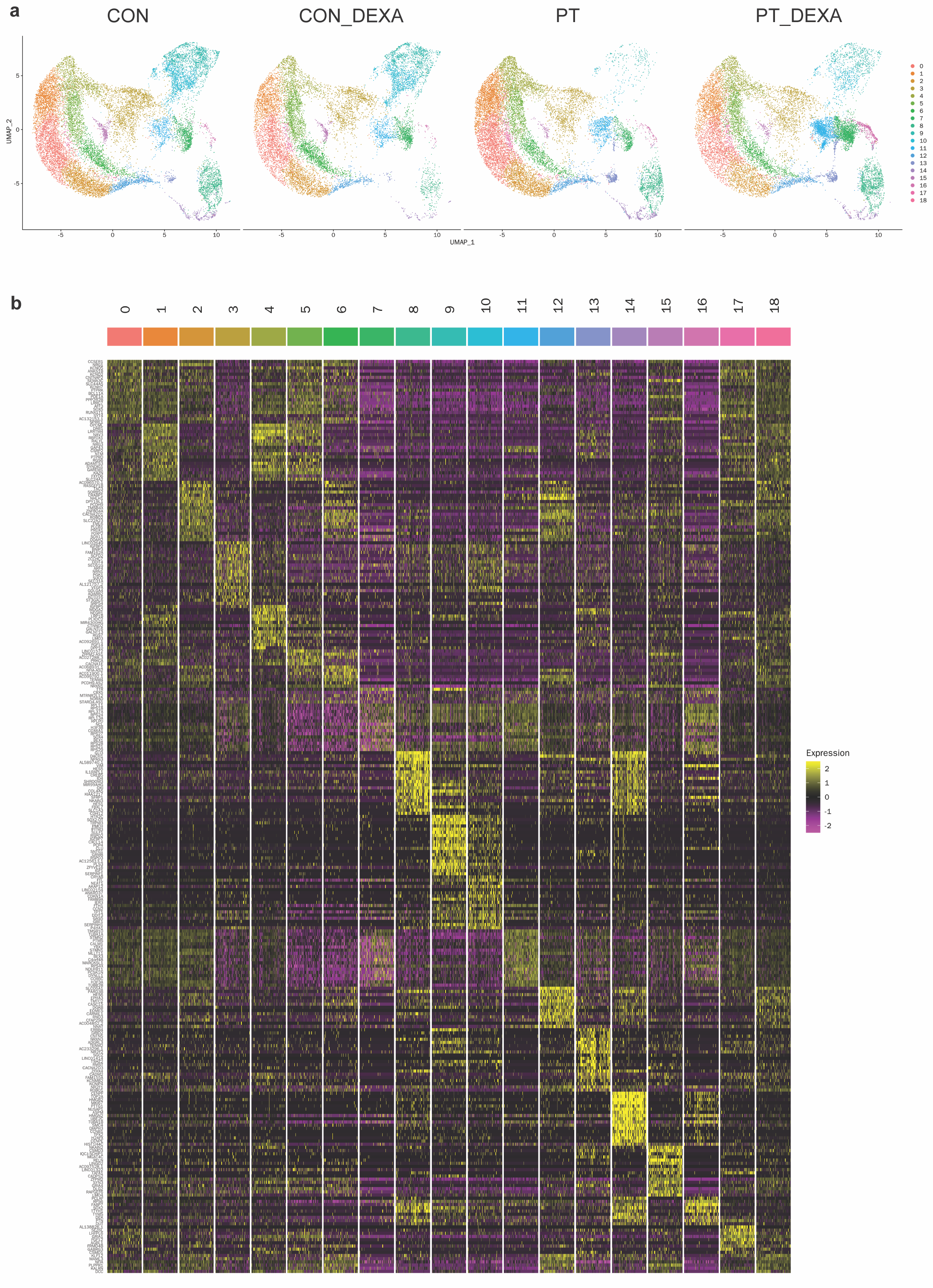
**

**Supplementary Figure 3. Single cell transcriptomic results of brain organoids of a patient and a control**

(A) Identified cell types through Seurat analysis from single-cell RNA sequencing of brain organoids and the distribution of cell types among brain organoids (n=3 for each group) from controls (CON) and patients (PT), both before and after dexamethasone (DMX) treatment.

(B) Heatmap showing the gene expression patterns for the top 20 genes in each cluster.

**
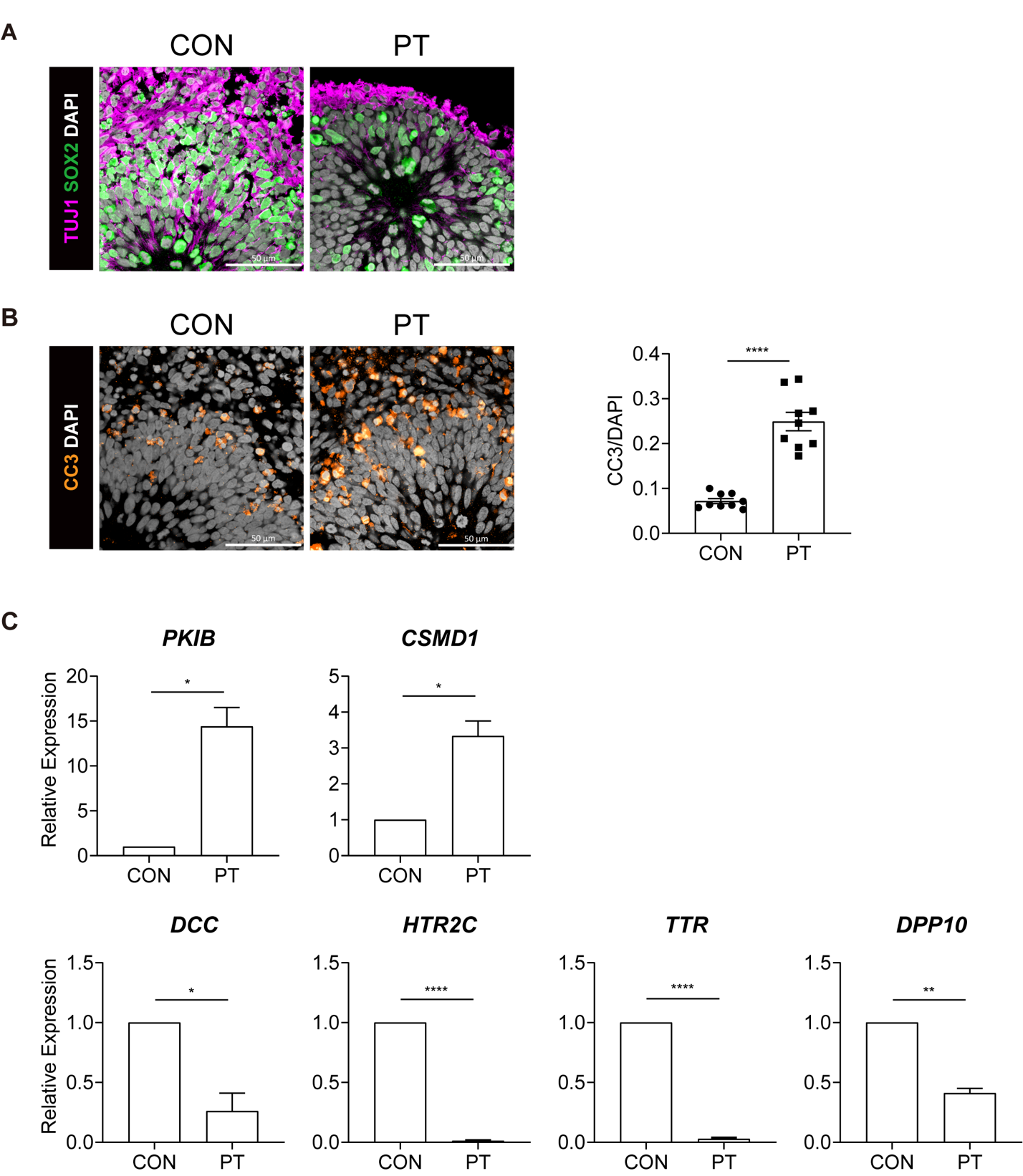
**

**Supplementary Figure 4. Characterization of neural differentiation, apoptosis, and gene expression changes in control (CON) and patient (PT) brain organoids**

(A) Immunocytochemistry (ICC) analysis for neural progenitor (SOX2⁺, green) and neuronal (TUJ1⁺, magenta) markers in CON and PT brain organoids at day 67. Nuclei were counterstained with DAPI (pseudocolored gray). Scale bar, 50 µm.

(B) ICC analysis for cleaved caspase-3 (CC3, pseudocolored orange) in day 67 CON and PT organoids. The proportion of CC3⁺ cells relative to total DAPI⁺ nuclei was quantified from three independent organoids, with three regions of interest (ROIs) analyzed per organoid. Data are shown as mean ± SEM. Statistical analysis was performed using an unpaired two-tailed t-test (****p < 0.0001). Scale bar, 50 µm.

(C) Quantitative PCR (qPCR) validation of differentially expressed genes (DEGs) identified by single-cell RNA sequencing (scRNA-seq) at day 60 of organoid differentiation. Upregulated genes in PT organoids (*PKIB, CSMD1*) and downregulated genes (*DCC, HTR2C, TTR, DPP10*) were validated. Data are represented as mean ± SEM (n = 2 organoids per group). Statistical significance was determined using an unpaired two-tailed t-test (*p < 0.05; **p < 0.01; ****p < 0.0001; ns, not significant).


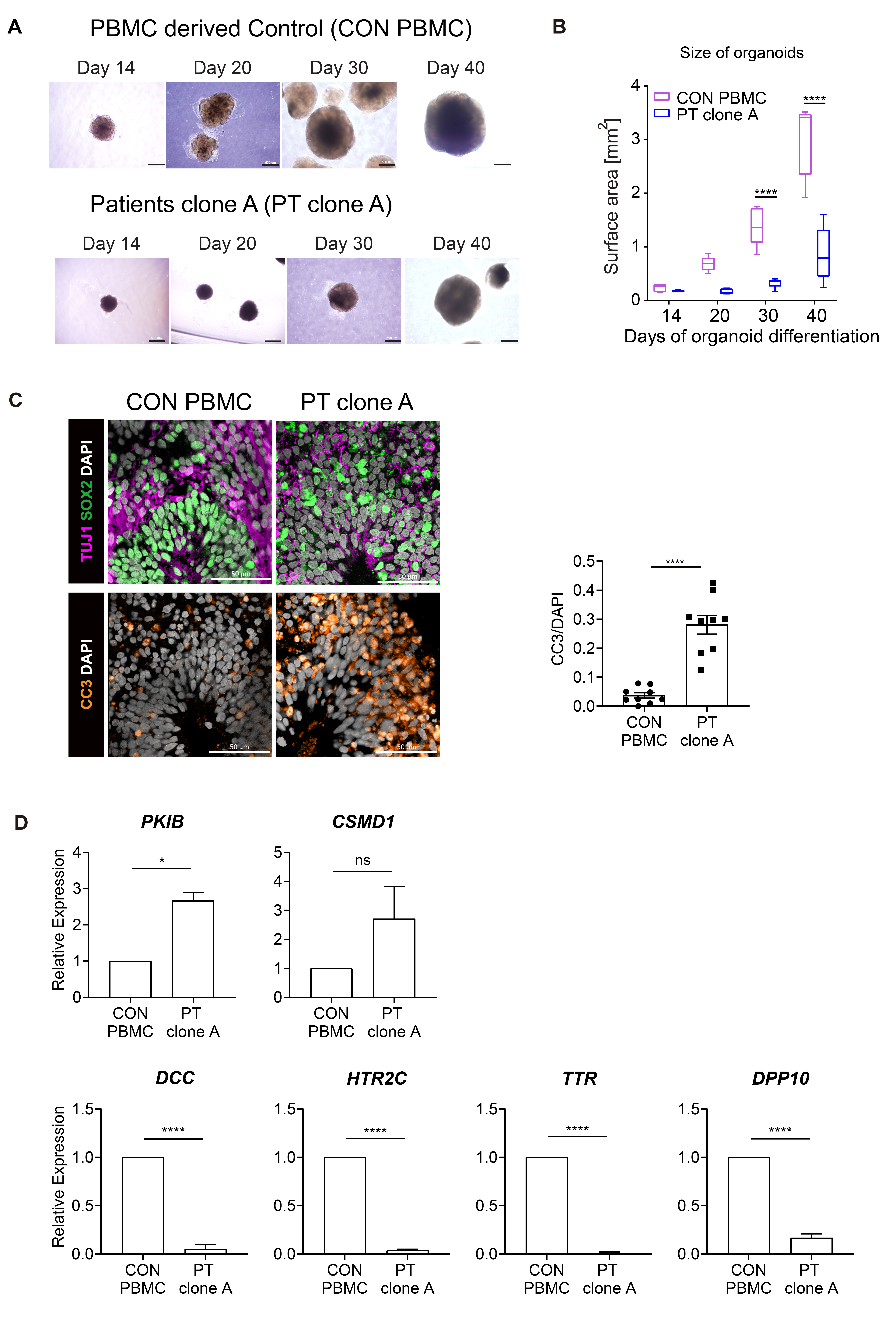


**Supplementary Fig. 5. Characterization of brain organoids differentiated from PBMC-derived control iPSCs (CON-PBMC) and patient iPSCs clone A (PT-clone-A).**

(A) Bright-field images of brain organoids generated from CON-PBMC and PT-clone-A iPSCs at days 14, 20, 30, and 40 of differentiation. Images were taken at the same magnification. Scale bars, 50 µm.

(B) Quantification of organoid surface area at days 14, 20, 30, and 40. Data represent mean ± SEM (n = 5 CON-PBMC organoids, n = 5 PT-clone-A organoids). Statistical analysis was performed using two-way ANOVA with Bonferroni’s multiple comparisons test (****p < 0.0001).

(C) ICC of day-40 organoids for neural progenitor marker SOX2 (green) and neuronal marker TUJ1 (magenta). Nuclei were counterstained with DAPI (gray). Representative images are shown for CON-PBMC and PT-clone-A organoids. Scale bars, 50 µm. CC3 (pseudocolored orange) staining was performed to assess apoptosis. The proportion of CC3⁺ cells relative to DAPI⁺ nuclei was quantified from three independent organoids, with three ROIs analyzed per organoid. Data are shown as mean ± SEM. Unpaired two-tailed t-test (****p < 0.0001).

(D) qPCR-based verification of scRNA-seq-identified genes at day 40 of organoid differentiation. Genes upregulated in PT organoids (*PKIB, CSMD1*) and downregulated genes (*DCC, HTR2C, TTR, DPP10*) shown in Supplementary Figure 4 were validated. Data are represented as mean ± SEM (n = 3 organoids per group). Statistical analysis was performed using an unpaired two-tailed t-test. Statistical significance was determined using an unpaired two-tailed t-test (*p < 0.05; ****p < 0.0001; ns, not significant).
